# Estimation of clinical trial success rates and related parameters

**DOI:** 10.1093/biostatistics/kxx069

**Published:** 2018-01-31

**Authors:** Chi Heem Wong, Kien Wei Siah, Andrew W Lo

**Affiliations:** 1 *MIT Computer Science and Artificial Intelligence Laboratory & Department of Electrical Engineering and Computer Science, Cambridge, MA 02139, USA and MIT Sloan School of Management and Laboratory for Financial Engineering, Cambridge, MA 02142, USA*; 2 *MIT Computer Science and Artificial Intelligence Laboratory & Department of Electrical Engineering and Computer Science, Cambridge, MA 02139, USA, MIT Sloan School of Management and Laboratory for Financial Engineering, Cambridge, MA 02142, USA, and AlphaSimplex Group, LLC, Cambridge, MA 02142, USA*

**Keywords:** Clinical phase transition probabilities, Clinical trial statistics, Probabilities of success

## Abstract

Previous estimates of drug development success rates rely on relatively small samples from databases curated by the pharmaceutical industry and are subject to potential selection biases. Using a sample of 406 038 entries of clinical trial data for over 21 143 compounds from January 1, 2000 to October 31, 2015, we estimate aggregate clinical trial success rates and durations. We also compute disaggregated estimates across several trial features including disease type, clinical phase, industry or academic sponsor, biomarker presence, lead indication status, and time. In several cases, our results differ significantly in detail from widely cited statistics. For example, oncology has a 3.4% success rate in our sample vs. 5.1% in prior studies. However, after declining to 1.7% in 2012, this rate has improved to 2.5% and 8.3% in 2014 and 2015, respectively. In addition, trials that use biomarkers in patient-selection have higher overall success probabilities than trials without biomarkers.

## 1. Introduction

The probability of success (POS) of a clinical trial is critical for clinical researchers and biopharma investors to evaluate when making scientific and economic decisions. Prudent resource allocation relies on the accurate and timely assessment of risk. Without up-to-date estimates of the POS, however, investors may misjudge the risk and value of drug development, leading to lost opportunities for both investors and patients.

One of the biggest challenges in estimating the success rate of clinical trials is access to accurate information on trial characteristics and outcomes. Gathering such data is expensive, time-consuming, and susceptible to error. Previous studies of success rates have been constrained by the data in several respects. Abrantes-Metz *and others* (2005) surveyed 2328 drugs using 3136 phase transitions (e.g., from Phase 1 to Phase 2 in the approval process), while DiMasi *and others* (2010) studied 1316 drugs from just 50 companies. In the landmark study of this area, Hay *and others* (2014) analyzed 7372 development paths of 4451 drugs using 5820 phase transitions. In two recent papers, Smietana *and others* (2016) computed statistics using 17 358 phase transitions for 9200 compounds, while Thomas *and others* (2016) used 9985 phase transitions for 7455 clinical drug development programs. In contrast, ClinicalTrials.gov, the clinical trial repository maintained by the National Institutes of Health (NIH), contains over 217 000 clinical trial entries submitted by various organizations as of July 1, 2016 (see www.clinicaltrials.gov). It is estimated that trained analysts would require tens of thousands of hours of labor to incorporate its full information manually to produce POS estimates.

In this article, we construct estimates of the POS and other related risk characteristics of clinical trials using 406 038 entries of industry- and non-industry-sponsored trials, corresponding to 185 994 unique trials over 21 143 compounds from Informa Pharma Intelligence’s Trialtrove and Pharmaprojects databases from January 1, 2000 to October 31, 2015. This is the largest investigation thus far into clinical trial success rates and related parameters. To process this large amount of data, we develop an automated algorithm that traces the path of drug development, infers the phase transitions, and computes the POS statistics in hours. In this article, we introduce the “path-by-path” approach that traces the proportion of development paths that make it from one phase to the next. In contrast, extant literature uses what we call the “phase-by-phase” approach, which estimates the POS from a random sample of observed phase transitions. Apart from the gains in efficiency, our algorithmic approach allows us to perform previously infeasible computations, such as generating time-series estimates of POS and related parameters.

We estimate aggregate success rates, completion rates (CRs), phase-transition probabilities, and trial durations, as well as more disaggregated measures across various dimensions such as clinical phase, disease, type of organization, and whether biomarkers are used. Before presenting these and other results, we begin by discussing our methodology and describing some features of our data set.

## 2. Data

We use Citeline data provided by Informa Pharma Intelligence, a superset of the most commonly used data sources that combines individual clinical trial information from Trialtrove and drug approval data from Pharmaprojects. In addition to incorporating multiple data streams, including nightly feeds from official sources such as ClinicalTrials.gov, Citeline contains data from primary sources such as institutional press releases, financial reports, study reports, and drug marketing label applications, and secondary sources such as analyst reports by consulting companies. Secondary sources are particularly important for reducing potential biases that may arise from the tendency of organizations to report only successful trials, especially those prior to the FDA Amendments Act of 2007, which requires all clinical trials to be registered and tracked via ClinicalTrials.gov. Our database contains information from both US and non-US sources.

The database encodes each unique quartet of trial identification number, drug, indication, and sponsor as a data point. As such, a single trial can be repeated as multiple data points. The trials range from January 1, 2000, to October 31, 2015, the latter being the date that we received the data set. After deleting 46 524 entries with missing dates and unidentified sponsors, and 1818 entries that ended before January 1, 2000, 406 038 data points remain. Of these, 34.7% (141 086) are industry sponsored and 65.3% (264 952) are non-industry sponsored. In our industry-sponsored analysis, we counted 41 040 development paths or 67 752 phase transitions after the imputation process. Figure S1 in Section A1 of the supplementary material available at *Biostatistics* online contains an illustrative sample of the data set and some basic summary information.

Some trials are missing end-dates due to the failure of their sponsors to report this information. Since these dates are required by our algorithm, we estimate them by assuming that trials lasted the median duration of all other trials with similar features. Only 14.6% (59 208) of the data points required the estimation of end-dates.

## 3. Modeling the drug development process

To avoid confusion and facilitate the comparison of our results with those in the extant literature, we begin by defining several key terms. A drug development program is the investigation of a particular drug for a single indication (see top diagram of Figure S2 of the supplementary material available at *Biostatistics* online). A drug development program is said to be in Phase $i$ if it has at least one Phase $i$ clinical trial. If a Phase $i$ clinical trial concludes and its objectives are met, this trial is said to be completed. If it is terminated prematurely for any reason, except in the case that it has positive results, the trial is categorized as failed. Conditioned on one or more trial(s) being completed, the sponsor can choose to either pursue Phase $i+1$ trials, or simply terminate development. If the company chooses the former option, the drug development program is categorized as a success in Phase $i$, otherwise, it will be categorized as terminated in Phase $i$. See Figure S2 (bottom) of the supplementary material available at *Biostatistics* online for an illustration. The POS for a given Phase $i$, denoted by POS$_{i,i+1}$, is defined as the probability that the drug development program advances to the next phase. The probability of getting a drug development program in Phase $i$ through to approval is denoted by POS$_{i,{\rm APP}}$. Hence the overall probability of success—moving a drug from Phase 1 to approval, which Hay *and others* (2014) calls the likelihood of approval (LOA)—is POS$_{1,{\rm APP}}$.

The proper interpretation of drug development programs from clinical trial data requires some understanding of the drug development process, especially in cases of missing data. This is particularly important for estimating a drug candidate’s POS$_{1,{\rm APP}}$, which is typically estimated by multiplying the empirical POS of Phase 1 (safety), 2 (efficacy for a given indication), and 3 (efficacy for larger populations and against alternatives) trials. If, for example, Phase 2 data are missing for certain approved drugs, the estimated POS$_{1,{\rm APP}}$ would be biased downward. Here, we take a different approach to estimating POSs.

Consider an idealized process in which every drug development program passes through Phase 1, 2, and 3 trials, in this order. This is plausible, since each of these stages involves distinct predefined tests, all of which are required by regulators in any new drug application (NDA). If we observe data for Phases 1 and 3 but not Phase 2 for a given drug-indication pair, our idealized process implies that there was at least one Phase 2 trial that occurred, but is missing from our data set. Accordingly, we impute the successful completion of Phase 2 in these cases. There exist some cases where Phase 2 trials are skipped, as with the recent example of Aducanumab (BIIB037), Biogen’s Alzheimer’s candidate, as reported by Root (2014). Since skipping Phase 2 trials is motivated by compelling Phase 1 data, imputing the successful completion of Phase 2 trials in these cases to trace drug development paths may not be a bad approximation. In addition, we make the standard assumption that Phase 1/2 and Phase 2/3 trials are to be considered as Phase 2 and Phase 3, respectively.

These assumptions allow us to more accurately reconstruct ‘drug development paths’ for individual drug-indication pairs, which in turn yield more accurate POS estimates. Let $n^j$ be the number of drug development paths with observed Phase $j$ trials, and $n^j_s$ be the number of drug development paths where we observe phase transitions of state $s$ of Phase $j$ (defined below).

\begin{equation*}
s = \begin{cases}
ip, & \mbox{if all the trials are in progress}\\
t, & \mbox{if the program failed to proceed to phase } i+1 \mbox{ (i.e., terminated)} \\
m, & \mbox{if the phase transition can be inferred to be missing} \\
\end{cases}
\end{equation*}

Equation 3.1 is the conservation law for drug development paths in Phase $j+1$.

(3.1) \begin{equation*}\label{eqn1} n^{j+1}=n^j + n_{m}^{j} - n_{ip}^{j} - n_{t}^{j} \qquad \forall j=1,2,3 \end{equation*}

The POS from any one state to the next, ${\rm POS}_{j,j+1}$, is thus the ratio of the number of drug development projects in Phase $j+1$, both observed and non-observed, to the number of drug development projects in Phase $j$, both observed and non-observed:

(3.2) \begin{equation*}\label{eqn2} {\rm POS}_{j,j+1}\text{(Path-by-Path)}=\frac{n^{j+1}}{{n^j} + n_{m}^{j} - n_{ip}^j} \end{equation*}

Given our model, we can now compute ${\rm POS_{1,APP}}$ by finding the proportion of development paths that made it from Phase 1 to Approval:

(3.3) \begin{equation*}\label{eqn3} {\rm {POS}_{1,APP}}\text{(Path-by-Path)}=\frac{n_{{}}^{\rm Approval}}{n_{{}}^{1}+n_{m}^{1}-n_{ip}^{1}-n_{ip}^{2}-n_{ip}^{3}} \end{equation*}

We term this the ‘path-by-path’ approach. In contrast, extant papers define the phase transition probability as the ratio of observed phase transitions to the number of observed drug development programs in Phase $i$ and multiply the individual phase probabilities to estimate the overall POS. We term this the ‘phase-by-phase’ approach, which we shall differentiate from the path-by-path computation by a superscript $p$ as follows:

(3.4) \begin{align*}\label{eqn4} {\rm POS}_{j,j+1}^p & =\frac{n^{j+1} - n_m^j} {n^j - n_{ip}^j}\\ \end{align*}

(3.5) \begin{align*}\label{eqn5} {\rm POS}_{\rm 1,APP}^{p} & =\prod\limits_{j\in \{1,2,3\}}{{\rm POS}_{j,j+1}^{p}} \end{align*}

Implicit in the path-by-path computation method is the assumption that we have relatively complete information about the trials involved in drug development programs. This is true of our data set, as we are analyzing relatively recent years where trial pre-registration is a prerequisite for publication in major medical journals and use of the studies as supporting evidence for drug applications.

However, this assumption breaks down when we look at short windows of duration, for example, in a rolling window analysis to estimate the change in the POS over time. In such cases, we default back to the ‘phase-by-phase’ estimation to get an insight into the trend. This is done by considering only those drug development programs with phases that ended between $t_1$ and $t_2$ in the computation of the POS.

(3.6) \begin{align*}\label{eqn6} {\rm POS}_{j,j+1}^{p} (t_1,t_2) & = \frac{n^{j+1}(t_1, t_2)-n_m^j(t_1, t_2)}{n^j(t_1, t_2) - n_{ip}^{j}(t_1, t_2)}\\ \end{align*}

(3.7) \begin{align*}\label{eqn7} {\rm POS}_{\rm 1,APP}^{p}(t_1, t_2) & = \prod\limits_{j\in \{1,2,3\}}{{\rm POS}_{j,j+1}^{p}}(t_1, t_2) \end{align*}

We further note that if no phase transitions are missing, the path-by-path and phase-by-phase methods should produce the same results, but the former will be more representative of actual approval rates if phase transitions are missing. We elaborate on this in Section A2 of the supplementary material available at *Biostatistics* online.

Given our development-path framework, we can compute the POS using an algorithm that recursively considers all possible drug-indication pairs and determines the maximum observed phase. Reaching Phase $i$ would imply that all lower phases were completed. To determine if a drug development program has been terminated in the last observed phase or is still ongoing, we use a simple heuristic: if the time elapsed between the end date of the most recent Phase $i$ and the end of our sample exceeds a certain threshold $t_i$, we conclude that the trial has terminated. Based on practical considerations, we set $t_i$ to be 360, 540, and 900 days for Phases 1, 2, and 3, respectively. For example, we assume that it takes approximately 6 months to prepare documents for an NDA filing after a Phase 3 trial has been completed. Since the FDA has a 6-month period to decide if it wishes to follow-up on a filing, and an additional 18 months to deliver a verdict, this places the overall time between Phase 3 and Approval to about 30 months, hence we set $t_3 = 900$ days. A pseudo-code for the algorithm is given in Figure S5 in Section A3 of the supplementary material available at *Biostatistics* online.

In summary, our algorithm allows us to impute missing trial data, and by counting the number of phase transitions, we can estimate the phase and overall POS.

## 4. Results

### 4.1. POS for all drugs and indications

Table 1 contains our estimates of the aggregate POS for each clinical phase across all indications. Corresponding estimates from the prior literature are also included for comparison. We find that 13.8% of all drug development programs eventually lead to approval, which is higher than the 10.4% reported by Hay *and others* (2014) and the 9.6% reported by Thomas *and others* (2016). The overall POS presented in this study, Hay *and others* (2014), and Thomas *and others* (2016) are much higher than the 1% to 3% that is colloquially seen as it is conditioned on the drug development program entering Phase 1. Our phase-specific POS estimates are higher in all phases. The largest increase is seen in POS$_{2,3}$, where we obtained a value of 58.3% compared to 32.4% in Hay *and others* (2014) and 30.7% in Thomas *and others* (2016). These differences may be due to our method of imputing missing clinical trials.

**Table 1.** *Comparison of the results of our article with previous publications using data from January 1, 2000, to October 31, 2015. We computed this using the algorithm shown in Fig. S5 in the Supplementary Material, which traces drug development programs and calculates the proportion of programs that advance from one phase to another*

	This study—all indications (industry)				This study— lead indications (industry)		Thomas *and others* (2016) —all indications		Hay *and others* (2014) —all indications		Hay *and others* (2014) —lead indications		DiMasi *and others* (2010) —lead indications	
Method	Path-by-Path		Phase-by-Phase		Path-by-Path		Phase-by-Phase		Phase-by-Phase		Phase-by-Phase		Phase-by-Phase	
	POS$_{i,i+1}$	POS$_{i,\text{APP}}$	POS$_{i,i+1}$	POS$_{i,\text{APP}}$	POS$_{i,i+1}$	POS$_{i,\text{APP}}$	POS$_{i,i+1}$	POS$_{i,\text{APP}}$	POS$_{i,i+1}$	POS$_{i,\text{APP}}$	POS$_{i,i+1}$	POS$_{i,\text{APP}}$	POS$_{i,i+1}$	POS$_{i,\text{APP}}$
Phase 1 to 2	66.4%	13.8%	38.8%	6.9%	75.8%	21.6%	63.2%	9.6%	64.5%	10.4%	66.5%	15.3%	71.0%	19.0%
Phase 2 to 3	58.3%	35.1%	38.2%	11.2%	55.9%	26.4%	30.7%	15.2%	32.4%	16.2%	39.5%	23.1%	45.0%	26.8%
Phase 3 to APP	59.0%	59.0%	59.0%	59.0%	70.0%	70.0%	49.6%	49.6%	50.0%	50.0%	58.4%	58.4%	60.0%	59.5%
Phase 1 to APP		13.8%		6.9%		21.6%		9.6%		10.4%		15.3%		19.0%
Number of drugs	15 102						Unknown		5820		4736		1316	
Years of source data (time-span)	2000–2015 (16 years)						2006–2015 (10 years)		2003–2011 (9 years)				1993–2009 (17 years)	
Number of companies	5764						1103		835				50	

Table 2 contains phase and overall POS estimates by therapeutic group. The overall POS (POS$_{1,\rm APP}$) ranges from a minimum of 3.4% for oncology to a maximum of 33.4% for vaccines (infectious disease). The overall POS for oncology drug development programs is about two-thirds the previously reported estimates of 5.1% in Thomas *and others* (2016) and 6.7% in Hay *and others* (2014).

**Table 2.** *The POS by therapeutic group, using data from January 1, 2000, to October 31, 2015. We computed this using the path-by-path method. SE denotes the standard error*

**All indications (industry)**								
	**Phase 1 to Phase 2**		**Phase 2 to Phase 3**			**Phase 3 to Approval**		**Overall**
		POS$_{1,2}$, %		POS$_{2,3}$, %	POS$_{2,\text{APP}}$, %		POS$_{3,\text{APP}}$, %	POS, %
**Therapeutic group**	Total paths	(SE, %)	Total paths	(SE, %)	(SE, %)	Total paths	(SE, %)	(SE, %)
Oncology	17 368	57.6	6533	32.7	6.7	1236	35.5	3.4
		(0.4)		(0.6)	(0.3)		(1.4)	(0.2)
Metabolic/	3589	76.2	2357	59.7	24.1	1101	51.6	19.6
Endocrinology		(0.7)		(1.0)	(0.9)		(1.5)	(0.7)
Cardiovascular	2810	73.3	1858	65.7	32.3	964	62.2	25.5
		(0.8)		(1.1)	(1.1)		(1.6)	(0.9)
CNS	4924	73.2	3037	51.9	19.5	1156	51.1	15.0
		(0.6)		(0.9)	(0.7)		(1.5)	(0.6)
Autoimmune/	5086	69.8	2910	45.7	21.2	969	63.7	15.1
Inflammation		(0.6)		(0.9)	(0.8)		(1.5)	(0.6)
Genitourinary	757	68.7	475	57.1	29.7	212	66.5	21.6
		(1.7)		(2.3)	(2.1)		(3.2)	(1.6)
Infectious disease	3963	70.1	2314	58.3	35.1	1078	75.3	25.2
		(0.7)		(1.0)	(1.0)		(1.3)	(0.8)
Ophthalmology	674	87.1	461	60.7	33.6	207	74.9	32.6
		(1.3)		(2.3)	(2.2)		(3.0)	(2.2)
Vaccines	1869	76.8	1235	58.2	42.1	609	85.4	33.4
(Infectious Disease)		(1.0)		(1.4)	(1.4)		(1.4)	(1.2)
Overall	41 040	66.4	21 180	58.3	35.1	7532	59.0	13.8
		(0.2)		(2.3)	(2.2)		(0.6)	(0.2)
All without	23 672	73.0	14 647	27.3	27.3	6296	63.6	20.9
oncology		(0.3)		(0.4)	(0.4)		(0.6)	(0.3)
**Lead indications (Industry)**								
	**Phase 1 to Phase 2**		**Phase 2 to Phase 3**			**Phase 3 to Approval**		**Overall**
		POS$_{1,2}$, %		POS$_{2,3}$, %	POS$_{2,\text{APP}}$, %		POS$_{3,\text{APP}}$, %	POS, %
**Therapeutic group**	Total paths	(SE, %)	Total paths	(SE, %)	(SE, %)	Total paths	(SE, %)	(SE, %)
Oncology	3107	78.7	1601	53.9	13.1	431	48.5	11.4
		(0.7)		(1.2)	(0.8)		(2.4)	(0.7)
Metabolic/	2012	75.2	1273	57.0	26.4	535	62.8	21.3
Endocrinology		(1.0)		(1.4)	(1.2)		(2.1)	(1.0)
Cardiovascular	1599	71.1	1002	64.9	34.1	473	72.3	26.6
		(1.1)		(1.5)	(1.5)		(2.1)	(1.2)
CNS	2777	75.0	1695	54.5	24.1	648	63.0	19.3
		(0.8)		(1.2)	(1.0)		(1.9)	(0.9)
Autoimmune/	2900	78.9	1862	48.7	24.3	659	68.6	20.3
Inflammation		(0.8)		(1.2)	(1.0)		(1.8)	(0.9)
Genitourinary	568	73.4	382	59.2	31.9	176	69.3	25.3
		(1.9)		(2.5)	(2.4)		(3.5)	(2.0)
Infectious Disease	2186	74.6	1326	58.0	34.3	594	76.6	26.7
		(0.9)		(1.4)	(1.3)		(1.7)	(1.1)
Ophthalmology	437	89.0	302	57.6	30.5	124	74.2	30.7
		(1.5)		(2.8)	(2.6)		(3.9)	(2.7)
Vaccines	881	75.8	567	57.1	40.4	269	85.1	31.6
(Infectious Disease)		(1.4)		(2.1)	(2.1)		(2.2)	(1.7)
Overall	16 467	75.8	10 010	55.9	26.4	3909	67.7	21.6
		(0.3)		(0.5)	(0.4)		(0.7)	(0.4)
All without	13 360	75.8	8409	29.0	29.0	3478	70.0	23.4
oncology		(0.4)		(0.5)	(0.5)		(0.8)	(0.4)

A significantly different pattern emerges when we consider the phase POS for lead indications. The overall POS (POS$_{1,\rm APP}$) increases when considering only lead indications, which is in line with the findings by Hay *and others* (2014). However, while we find an increase in the POS for Phase 1 (POS$_{1,2}$) and Phase 3 (POS$_{3,{\rm APP}}$), we find a decrease in the POS for Phase 2 (POS$_{2,3}$) when looking only at lead indications. The POS for lead indications may be lower than the POS for all indications if a company initiates clinical trials for many indications, and most of them move on to the next phase. Conversely, the POS for lead indications may be higher if many of the initiated clinical trials for the same drug fail. The practice of initiating clinical trials for multiple indications using the same drug is prevalent in the industry, as documented in Table S2 in Section A5 of the supplementary material available at *Biostatistics* online. The relative performance of the various therapeutic groups remains the same when considering only lead indications, with oncology remaining the lowest performing group at 11.4% for POS$_{1,\rm APP}$. Finally, the overall POS for individual therapeutic groups when considering only lead indications shows mixed directions in comparison to the respective overall POS specific to the indication.

### 4.2. POS of trials with biomarkers

As the use of biomarkers to select patients, enhance safety, and serve as surrogate clinical endpoints has become more common, it has been hypothesized that trials using biomarkers are more likely to succeed. We test this hypothesis by comparing the POS of drugs with and without biomarkers.

We perform two separate analyses. In the first, we investigate the use of biomarkers only for patient selection, as did Thomas *and others* (2016). In the second, we expand the definition of a biomarker trial to include those trials with the objective of evaluating or identifying the use of any novel biomarkers as indicators of therapeutic efficacy or toxicity, in addition to those that use biomarkers for patient selection.

In our database, only 7.1% of all drug development paths that use biomarkers use them in all stages of development. As such, we adopt the phase-by-phase approach instead of using the path-by-path approach. This is done by modifying Algorithm 1 (see Figure S5 of supplementary material available at *Biostatistics* online) to increment counts only if there exists a biomarker trial in that phase. Furthermore, as 92.3% of the trials using biomarkers in our database are observed only on or after January 1, 2005, we do not include trials before this date to ensure a fair comparison of the POS between trials that do and do not use biomarkers.

Table 3 shows only trials that use biomarkers to stratify patients. As can be seen, there is substantial variation in the use of biomarkers across therapeutic areas. Biomarkers are seldom used outside of oncology. Trials using biomarkers exhibit almost twice the overall POS (POS$_{1,\rm APP}$) compared to trials without biomarkers (10.3% vs. 5.5%). While the use of biomarkers in the stratification of patients improves the POS in all phases, it is most significant in Phases 1 and 2. (We caution against over-interpreting the results for therapeutic areas outside oncology due to their small sample size.) These findings are similar in spirit to the analysis by Thomas *and others* (2016), which also found substantial improvement in the overall POS when biomarkers were used.

**Table 3.** *POS of drug development programs with and without biomarkers, using data from January 1, 2005, to October 31, 2015, computed using the phase-by-phase method. These results consider only trials that use biomarkers in patient stratification. Since for the majority of trials using biomarkers (92.3%) their status is observed only on or after January 1, 2005, the choice of the time period is to ensure a fair comparison between trials using and not using biomarkers. SE denotes standard error*

**Biomarkers**												
		**Phase 1 to Phase 2**			**Phase 2 to Phase 3**			**Phase 3 to approval**			**Overall**	
**Therapeutic group**		Total phase transitions	POS$_{1,2}$, %	(SE, %)	Total phase transitions	POS$_{2,3}$, %	SE, %	Total phase transitions	POS$_{3,\text{APP}}$, %	(SE, %)	POS, %	(SE, %)
Oncology	No biomarker	9349	28.0	(0.5)	4773	17.4	(0.5)	1159	33.6	(1.4)	1.6	(0.2)
	With biomarker	1136	43.5	(1.5)	742	38.8	(1.8)	77	63.6	(5.5)	10.7	(1.9)
	All	10 485	29.7	(0.4)	5515	20.3	(0.5)	1236	35.5	(1.4)	2.1	(0.2)
Metabolic/	No biomarker	1532	44.5	(1.3)	1438	33.9	(1.2)	1086	52.0	(1.5)	7.9	(0.8)
endocrinology	With biomarker	7	57.1	(18.7)	2	50.0	(35.4)	15	20.0	(10.3)	5.7	(13.9)
	All	1539	44.6	(1.3)	1440	34.0	(1.2)	1101	51.6	(1.5)	7.8	(0.8)
Cardiovascular	No biomarker	1241	39.6	(1.4)	1027	37.9	(1.5)	962	62.2	(1.6)	9.3	(1.0)
	With biomarker	7	85.7	(13.2)	5	100.0	(0.0)	2	100.0	(0.0)	85.7	(13.2)
	All	1248	39.9	(1.4)	1032	38.2	(1.5)	964	62.2	(1.6)	9.5	(1.0)
CNS	No biomarker	2181	40.4	(1.1)	2050	30.2	(1.0)	1141	51.1	(1.5)	6.2	(0.6)
	With biomarker	42	54.8	(7.7)	42	28.6	(7.0)	15	53.3	(12.9)	8.3	(6.4)
	All	2223	40.7	(1.0)	2092	30.2	(1.0)	1156	51.1	(1.5)	6.3	(0.6)
Autoimmune/	No biomarker	2506	38.9	(1.0)	2106	25.4	(0.9)	964	63.7	(1.5)	6.3	(0.6)
inflammation	With biomarker	9	55.6	(16.6)	14	35.7	(12.8)	5	60.0	(21.9)	11.9	(16.8)
	All	2515	39.0	(1.0)	2120	25.5	(0.9)	969	63.7	(1.5)	6.3	(0.6)
Genitourinary	No biomarker	359	34.3	(2.5)	287	28.9	(2.7)	212	66.5	(3.2)	6.6	(1.5)
	With biomarker	5	80.0	(17.9)	0	N.A.	N.A.	0	N.A.	N.A.	N.A.	N.A.
	All	364	34.9	(2.5)	287	28.9	(2.7)	212	66.5	(3.2)	6.7	(1.5)
Infectious disease	No biomarker	1961	39.7	(1.1)	1453	34.7	(1.2)	1069	75.1	(1.3)	10.4	(0.9)
	With biomarker	6	66.7	(19.2)	27	44.4	(9.6)	9	100.0	(0.0)	29.6	(16.8)
	All	1967	39.8	(1.1)	1480	34.9	(1.2)	1078	75.3	(1.3)	10.5	(0.9)
Ophthalmology	No biomarker	180	52.2	(3.7)	274	34.7	(2.9)	207	74.9	(3.0)	13.6	(2.8)
	With biomarker	1	0.0	(0.0)	3	33.3	(27.2)	0	N.A.	N.A.	N.A.	N.A.
	All	181	51.9	(3.7)	277	34.7	(2.9)	207	74.9	(3.0)	13.5	(2.8)
Vaccines	No biomarker	733	40.8	(1.8)	761	32.9	(1.7)	609	85.4	(1.4)	11.4	(1.3)
(infectious disease)	With biomarker	0	N.A.	N.A.	5	0.0	(0.0)	0	N.A.	N.A.	N.A.	N.A.
	All	733	40.8	(1.8)	766	32.6	(1.7)	609	85.4	(1.4)	11.4	(1.3)
Overall	No biomarker	20 042	34.7	(0.3)	14 169	26.8	(0.4)	7409	59.0	(0.6)	5.5	(0.2)
	With biomarker	1213	44.5	(1.4)	840	38.6	(1.7)	123	60.2	(4.4)	10.3	(1.6)
	All	21 255	35.2	(0.3)	15 009	27.4	(0.4)	7532	59.0	(0.6)	5.7	(0.2)

However, when we expanded the definition of a biomarker trial to include trials with the objective of evaluating or identifying the use of any novel biomarker as an indicator of therapeutic efficacy or toxicity, in addition to the selection of patients, we obtained significantly different results (see Table S3 in Section A6 of the supplementary material available at *Biostatistics* online). Instead of finding a huge increase in the overall POS, we find no significant difference. It may be that trials that attempt to evaluate the effectiveness of biomarkers are more likely to fail, leading to a lower overall POS compared to trials that only use biomarkers in patient stratification. Comparison of the two tables shows that new biomarkers are being evaluated in all therapeutic areas.

We provide a more detailed analysis of the differences between our analysis and Thomas *and others* (2016) in Section A7 of the supplementary material available at *Biostatistics* online.

### 4.3. POS of orphan drugs trials

Table 4 contains POS estimates for drugs that treat rare diseases, also known as ‘orphan drugs’. The classifications for rare diseases are obtained from both EU and US rare disease resources: OrphaNet and NIH GARD. Rare diseases may belong to any therapeutic group, and the computation of the statistics for orphan drugs is identical to that used for the trials in Table 2.

**Table 4.** *The POS of orphan drug development programs. We computed the results using the path-by-path method. While we used the entire data set from January 1, 2000, to October 31, 2015, it has to be noted that there are only 3548 data points relating to orphan drugs, with the majority (95.3%) of the trials’ statuses observed on or after January 1, 2005. SE denotes standard error*

**Orphan drugs (industry, all indications)**								
	**Phase 1 to Phase 2**		**Phase 2 to Phase 3**			**Phase 3 to Approval**		**Overall**
**Therapeutic group**	Total paths	POS$_{1,2}$, % (SE, %)	Total paths	POS$_{2,3}$, % (SE, %)	POS$_{2,\text{APP}}$, % (SE, %)	Total paths	POS$_{3,\text{APP}}$, % (SE, %)	POS, % (SE, %)
Oncology	1245	72.0	535	39.4	2.8	104	14.4	1.2
		(1.3)		(2.1)	(0.5)		(3.4)	(0.3)
Metabolic/endocrinology	89	84.3	45	66.7	31.1	18	77.8	15.7
		(3.9)		(7.0)	(4.9)		(9.8)	(3.9)
Cardiovascular	115	69.6	58	77.6	43.1	30	83.3	21.7
		(4.3)		(5.5)	(4.6)		(6.8)	(3.8)
CNS	160	85.0	96	56.3	8.3	25	32.0	5.0
		(2.8)		(5.1)	(2.2)		(9.3)	(1.7)
Autoimmune/inflammation	228	76.3	114	57.0	8.8	32	31.3	4.4
		(2.8)		(4.6)	(1.9)		(8.2)	(1.4)
Genitourinary	14	100.0	13	46.2	38.5	6	83.3	35.7
		(0.00)		(13.8)	(13.0)		(15.2)	(12.8)
Infectious disease	157	89.2	104	53.8	28.8	39	76.9	19.1
		(2.5)		(4.9)	(3.6)		(6.7)	(3.1)
Ophthalmology	19	73.7	7	71.4	0.0	0	N.A	N.A
		(10.1)		(17.1)	(0.00)		N.A	N.A
Vaccines (infectious disease)	57	89.5	43	53.5	51.2	22	100.0	38.6
		(4.06)		(7.6)	(6.6)		(0.0)	(6.4)
Overall	2084	75.9	1015	48.8	12.7	276	46.7	6.2
		(0.9)		(1.6)	(0.7)		(3.0)	(0.5)
All except oncology	839	81.5	480	59.2	23.8	172	66.3	13.6
		(1.3)		(2.2)	(1.5)		(3.6)	(1.2)

Broadly speaking, orphan drug development has significantly lower success rates, with only 6.2% of drug development projects reaching the market. Comparing these results against those for all drug development, we see that, while the Phase 1 POS increases from 66.4% to 75.9%, the Phase 2 and Phase 3 success rates fall from 58.3% to 48.8% and from 59.0% to 46.7%, respectively, leading to a decline in the overall POS.

Our data reveal that most orphan drug trials are in oncology. Our overall POS of 6.2% is much lower than the 25.3% reported in Thomas *and others* (2016). This discrepancy can be attributed to their identification of only non-oncology indications as ‘rare diseases’ and their use of the phase-by-phase method of computing the POS. Our estimated orphan drug POS increases to 13.6% after excluding all oncology indications from the calculations, which is more in line with the findings of Thomas *and others* (2016).

### 4.4. POS over time

Many observers in both industry and academia believe that the success rate of clinical drug development projects has fallen over the past decade. We attempt to evaluate this belief quantitatively by computing the sample phase success rates for the years between 2005 and 2015 using 3-year rolling windows to capture time variation while smoothing estimation errors. We define the 3-year window to be January 1 in year $t-2$ to December 31 in year $t$, with the exception of the last window, which terminates on October 31, 2015, the day we received the snapshot of the data. Caution must be exercised in interpreting the results for 2015, which very likely overestimate the true success rates due to boundary effects. As insufficient time has passed to allow our algorithm to conclude that a trial has failed, we obtain a smaller denominator in our computation of probabilities, translating to an upward bias in the success rates for that year.

**Fig. 1.**
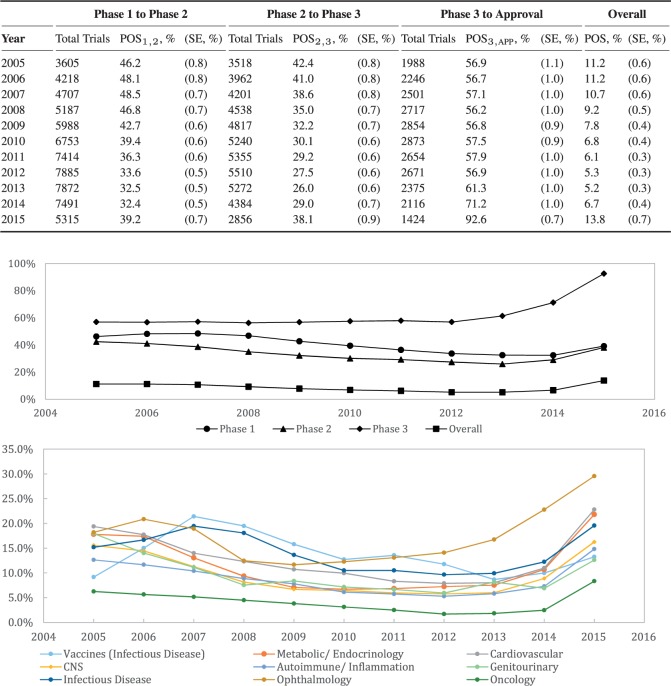
The POS over the period of January 1, 2005, to October 31, 2015, computed using a 3-year rolling window from January 1 in year $t-2$ to December 31 in year $t$, with the exception of the last window, which terminates on October 31, 2015. This POS is computed using the phase-by-phase method, our adaptation of Hay *and others* (2014)’ methodology, which reports the proportion of phase transitions that advances to the next phase. The algorithm from Figure S5 in the Supplementary Material is not used, as it would overestimate the phase success if applied to a short window. The result for 2015 has to be treated with caution, as boundary effects increase the success rates artificially.

We find that the overall success rate for all drug development programs did decrease between 2005 (11.2%) and 2013 (5.2%), as anecdotal reports suggest. However, this decline reversed after 2013 (see Figure 1). The overall success rate is mainly driven by changes in POS$_{1,2}$ and POS$_{2,3}$. The timing of the upward trend coincides with the time period during which the FDA has been approving more novel drugs, compared to the historical mean (see U.S. Food and Drug Administration, Center for Drug Evaluation and Research, 2016). Our results are not directly comparable with Smietana *and others* (2016) because it used a different aggregation method and considered only lead indications.

The overall POSs across the different therapeutic groups move in tandem over time. There are some minor deviations, such as the POS of drugs and vaccines for infectious diseases increasing between 2005 and 2007. Nonetheless, these results suggest that there is a systemic factor driving the trends over time. Numerical results of our analysis are provided in Section A8 of the supplementary material available at *Biostatistics* online.

### 4.5. Other results

We quantify other aspects of clinical drug development including CRs, duration, and POS for non-industry-sponsored trials, which we summarize here (see Sections A9 through A14 in the supplementary material available at *Biostatistics* online for details).

We find that the average number of Phase 1, Phase 2, Phase 3, and Phase 4 trials per drug development path are 1.7, 2.0, 2.8, and 3.2, respectively. The high number of Phase 4 trials per development path is surprising, as it indicates that many approved drugs have to conduct massive post-marketing studies to identify and evaluate long-term side effects. This may potentially increase drug development costs and lower the profitability of the drugs in the long run.

The CR at Phase $i$ refers to the proportion of Phase $i$ trials that are tagged as completed. Our data show that the CRs for all clinical trials are 91.4%, 81.1%, 84.9%, and 87.2% for Phases 1 through 4, respectively. While Phase 3 trials are often riskier and costlier, they have a higher CR than Phase 2 trials. This may be because Phase 3 trials are given higher priority within organizations. Oncology trials have lower CRs across all phases, with only 73.9% of the trials reaching completion. This points to a possible bottleneck in the development of oncology drugs.

Trial length is a key determinant of the financial risk and reward of drug development projects. We provide updated estimates for the duration of clinical trials using our data set. We find that the median clinical trial durations are 1.6, 2.9, and 3.8 years, for trials in Phases 1, 2, and 3, respectively. Our findings for Phase 3 are higher than Martin *and others* (2017), but lower for Phase 1. The clinical cycle times for Phase 2 trials are similar. By summing up the individual durations across Phases 1 through 3 and across therapeutic area, we find that the median time spent in the clinic ranged from 5.9 to 7.2 years for non-oncology trials, but the median duration for oncology trials was 13.1 years. This suggests higher risks in oncology projects and may explain their lower approval rate.

Similar to Abrantes-Metz *and others* (2005), we examine whether there is a difference between trials that fail to transition to the next phase of drug development (‘terminated’) and those that transition successfully (‘advanced’). Terminated Phase 2 trials tend to conclude 8.1 months earlier than advanced Phase 2 trials. However, terminated Phase 3 trials concluded about 3.2 months after advanced Phase 3 trials. The difference between terminated and advanced Phase 1 trials is insignificant. By constructing a time series using 5-year rolling windows, we see that these differences (or lack thereof) have remained constant over time.

The CRs for non-industry sponsored trials vs. industry-sponsored trials are higher for Phases 2 and 4, virtually identical for Phase 3, and lower for Phase 1. This mixed result suggests that synergies between industry and non-industry organizations can be exploited through collaboration. To evaluate this intuition, we compute the POS of drug development projects conditioned on the number of non-industry partners and find an 11.3 percentage point increase in the POS when non-industry partners are involved. This result extends the findings by Danzon *and others* (2005), underscoring the benefits of greater collaboration between the pharmaceutical industry and organizations outside the industry.

## 5. Robustness test

In this article, we attempt to use trial data to trace every drug/indication/sponsor triplet from first trial to last. While our method is arguably more accurate than earlier ones, it faces other issues such as the need to process machine-readable trial information using heuristics, and deal with corrupted and missing data by interpolation and estimation, which may introduce errors of their own. We perform two experiments to test the robustness and stability of our algorithm across different time windows and data sets.

In our first experiment, we attempt to replicate Thomas *and others* (2016) by using only data between 2006 and 2015. We find that the POS from the truncated sample differs from the full sample by less than 2.1 percentage points for all therapeutic groups, while the overall POS is 0.6 percentage points lower than the overall POS of the full sample. Compared to Thomas *and others* (2016), we find that our phase POS is higher in Phases 1 and 2, but lower in Phase 3, due to our use of the path-by-path method for calculating the POS. Our 13.8% overall POS is higher than their 9.6%.

In our second experiment, we run our algorithm using only data tagged as originating from ClinicalTrials.gov. The computed success rates are comparable to those from our original data set, with deviations of less than 2.1 percentage points despite having approximately 30% fewer data points. This indicates that our algorithm produces similar results even when a different data set is used. Details of our robustness results are provided in Section A15 of the supplementary material available at *Biostatistics* online.

## 6. Discussion

Compared with Hay *and others* (2014) and Thomas *and others* (2016), we obtained higher POS estimates for all phases. Our numbers will result in a lower estimated drug development cost, especially in Phase 3, where the cost of conducting a trial dominates those of other phases.

Looking at the trend of POS over time, we see that the there is a decrease between 2005 and 2013, and an increase thereafter. There are many possible reasons for the uptick in the later years. As some observers have suggested, companies may have been more careful with licensing compounds and gotten better at identifying potential failures (see Smietana *and others*, 2016), thus leading to higher productivity. We propose two other possible reasons for the trend. Firstly, the increased use of biomarkers in recent years could have contributed to the trend by allowing companies to target drugs at patients who are more likely to produce a positive response. Secondly, it is also possible that we are simply observing a new wave of medical breakthroughs, such as in the area of immunotherapy; for example, in the short span of just 11 months between July 2014 and June 2015, Nivolumab was approved on five different occasions for various indications in multiple markets.

We found that trials using biomarkers for patient stratification have higher success rates, especially in the area of oncology. Given the active development of biomarkers for the area of oncology, we expect that the dismal approval rates of oncology will improve. However, our expectations are tempered by the fact that the median time to completion for oncology trials is twice as long as for non-oncology trials, signifying increased cost and risk.

We believe these revised estimates of the success rates of clinical trials will provide greater risk transparency to drug developers, investors, policymakers, and patients. We hope that with this information, all stakeholders in the health care ecosystem will be able to make more informed decisions regarding the design and implementation of clinical trials.

## Supplementary Material

Supplementary Data
