# Corrigendum: Estimation of clinical trial success rates and related parameters

**DOI:** 10.1093/biostatistics/kxy072

**Published:** 2018-11-14

**Authors:** Chi Heem Wong, Kien Wei Siah, Andrew W Lo

**Affiliations:** 1 MIT Computer Science and Artificial Intelligence Laboratory & Department of Electrical Engineering and Computer Science, Cambridge, MA, USA; 1a MIT Sloan School of Management and Laboratory for Financial Engineering, Cambridge, MA, USA; 2b AlphaSimplex Group, LLC, Cambridge, MA, USA

*Biostatistics* (2019) **20**, 2, *pp*. 273–286

doi:10.1093/biostatistics/kxx069

In the original article, there were errors in Tables 1 and 2, specifically Phase 2 to 3 and Phase 3 to APP in Table 1, and Phase 2 to 3 columns in Table 2. Due to a formatting issue in Excel, incorrect numbers were reported originally.

There was a mis-match in the data between the pdf and the HTML article which has now been rectified. The tables have now been corrected. The author and Publisher apologizes for these errors.

## Supplementary Material

Supplementary Material

