# Supplementary material for: Estimation of clinical trial success rates and related parameters
Source: Biostatistics. 2018 Jan 31;20(2):273–86. doi: 10.1093/biostatistics/kxx069 (PMC6409418; doi:10.1093/biostatistics/kxx069)
Supplement: Supplementary Data [file kxx069_supp.pdf]

Supplementary Material for ‘Estimation of Clinical Trial Success Rates and Related Parameters’

A1. DATA

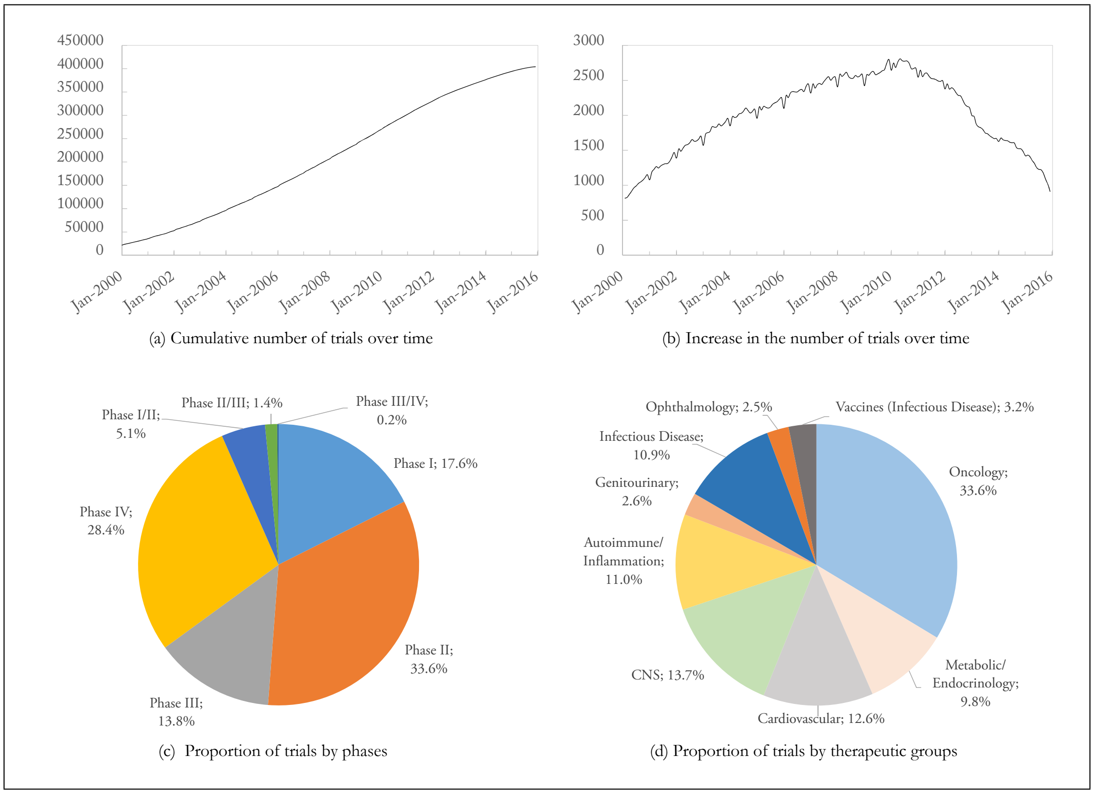

Fig. S1. Summary of the entire dataset of 406,038 data points, consisting of 185,994 unique trials. Of these, 34.7% are industry-sponsored (n=141,086) and the remaining 65.3% are non-industry sponsored (n=264,952). The trials span from January 1, 2000, to October 31, 2015.

TrialID	Therapeutic Area	Drug Name	Phase	Disease Type	Start Date	End Date	Sponsor
48391	Autoimmune/ Inflammation	Loratadine	I/2	Allergic Rhinitis	NULL	2003-06-07	(Other Hospital/ Academic/ Medical Center)
70538	Autoimmune/ Inflammation	Loratadine	3	Allergic Rhinitis	NULL	2007-09-18	(Other Hospital/ Academic/ Medical Center)
100378	Autoimmune/ Inflammation	Loratadine	3	Asthma	NULL	2008-10-29	Merck & Co.
122164	Autoimmune/ Inflammation	Loratadine	4	Allergic Rhinitis	2010-01-01	2012-03-01	(Other Hospital/ Academic/ Medical Center)
151465	CNS	Loratadine	3	Pain (nociceptive)	2011-05-01	2014-05-14	Cancer and Leukemia Group B (CALGB)
153368	Autoimmune/ Inflammation	Loratadine	I	Asthma	NULL	2006-07-01	(Other Hospital/ Academic/ Medical Center)

Table S1. Sample of Citeline data entries. Our algorithm processes such data to identify drug development programs and compute their statistics.

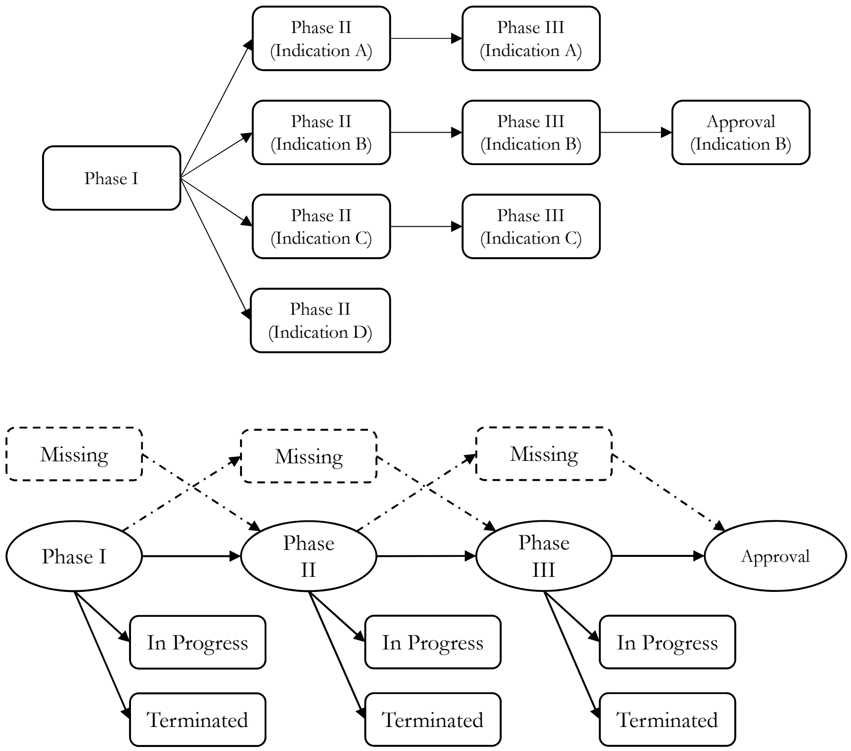

Fig. S2. (Top) We define a drug development path as the development of a drug for a specific indication. The diagram illustrates 4 drug development paths for a single drug. (Bottom) Observed and unobserved states in a drug development program, from Phase 1 to Approval. A drug development program is in Phase i if it has at least one trial in Phase i.

A2. Path-by-path vs. phase-by-phase

This paper uses the path-by-path method of computing the probability of success, where we identify all the drug development paths before computing the proportion of paths that make it through from Phase 1 to approval. In contrast, the phase-by-phase method computes the proportion of observed phase transitions from one phase to the next before multiplying the individual probabilities in each stage to produce the overall probability of success.

It is not uncommon for datasets to contain missing data points. For example, for some drugs and indications, we observe Phase 1 trials and Phase 3 trials, but not Phase 2 trials. This may occur because there is an error in data collection and data processing, or for other reasons.

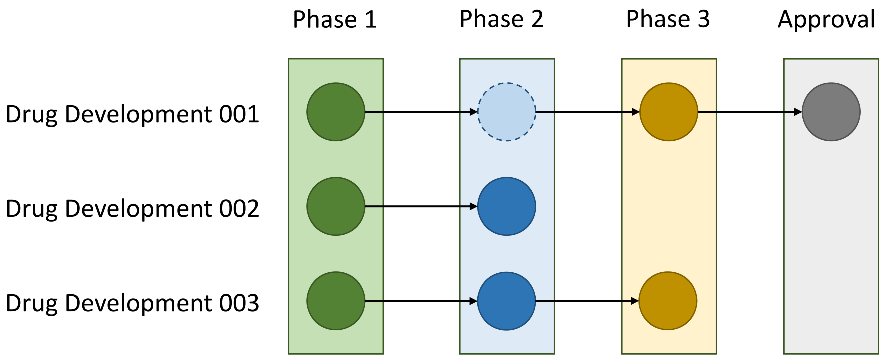

Fig. S3. In this example, we do not observe any Phase 2 trials for Drug Development 001. Our idealized model imputes the phase for the drug development and our 'path-by-path' method computes $\mathrm{POS}_{1,2}$, $\mathrm{POS}_{2,3}$, $\mathrm{POS}_{3,APP}$, and $\mathrm{POS}_{1,APP}$ to be 1, $\frac{2}{3}$, $\frac{1}{2}$ and $\frac{1}{3}$, respectively. In contrast, the 'phase-by-phase' method does not impute the phase and will compute $\mathrm{POS}_{1,2}$, $\mathrm{POS}_{2,3}$, $\mathrm{POS}_{3,APP}$, and $\mathrm{POS}_{1,APP}$ to be 1, $\frac{1}{2}$, $\frac{1}{2}$, and $\frac{1}{4}$, respectively.

We treat these cases as successes in our methodology. While we acknowledge that this may produce higher success rates for Phase 1 and Phase 2 trials, we find it only logical to include these 'missing' data points, as they definitely must have occurred in a development path. (We give an example of how the phase-by-phase method underestimates the POS in Figure S3.) In addition, we perform Monte Carlo simulations to demonstrate the impact of ignoring 'missing' phase transitions. Setting the $\mathrm{POS}_{1,2}$, $\mathrm{POS}_{2,3}$, and $\mathrm{POS}_{3,App}$ to be 0.5, we generate 1,000 drug development paths randomly and corrupt them to simulate missing phase transitions. We then run the phase-by-phase and path-by-path computations on the simulated data. As can be seen in Figure S4, which plots the means of 1,000 such runs, the path-by-path method accurately estimates the POS, while the phase-by-phase method underestimates the POS.

However, the path-by-path approach is not suitable in analyzing instances where one does not have the full information about the drug development programs, such as a rolling-window computation where the time window is much shorter than the complete drug development period (typically around a decade). This is because our algorithm aggressively imputes the 'missing' phase transitions when it is given only a snippet of information. We give a fictitious example to

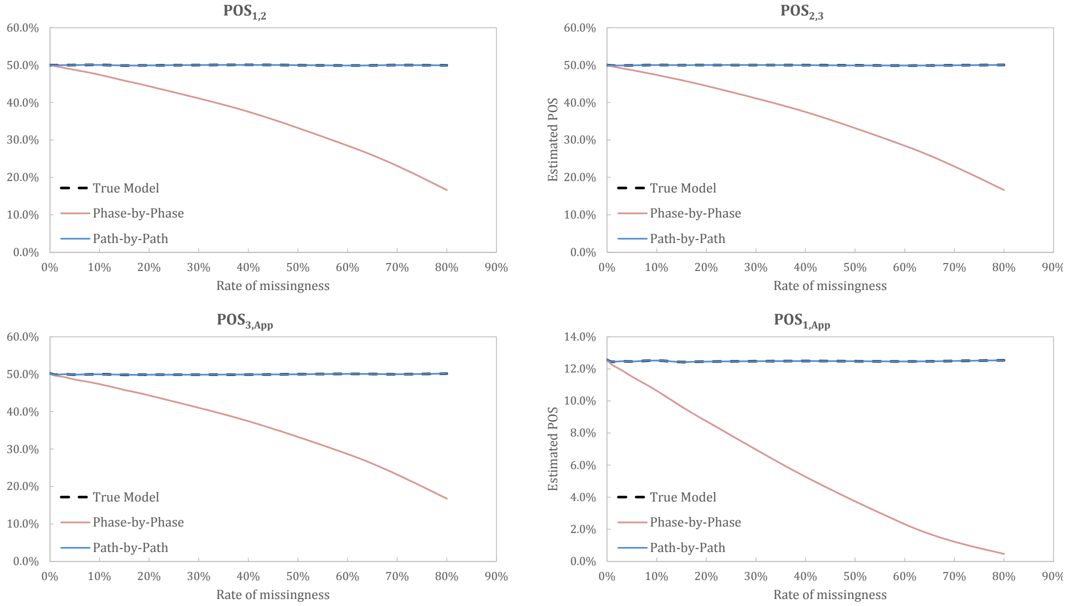

Fig. S4. Simulations of the computed POS using the phase-by-phase and path-by-path approaches. Results shown are the mean of 1,000 runs of 1,000 simulated drug paths with randomly corrupted phase transitions. The phase-by-phase approach consistently underestimates the POS in the presence of missing phase transitions.

illustrate this point.

Consider the following fictitious drug development program X.

Drug Development Program X

Phase	Start Date	End Date
1	Jan 2000	Jun 2000
2	Feb 2001	July 2003
3	Mar 2004	Dec 2007

The output of the different computation methods for the various 3-year time windows is as follows:

Time Window	Observed Phase	Path-by-Path	Phase-by-Phase
Jan 2000 to Dec 2002	1	Phase 1 completed	Phase 1 completed
Jan 2001 to Dec 2003	2	Phases 1 & 2 completed	Phase 2 completed
Jan 2002 to Dec 2004	2	Phases 1 & 2 completed	Phase 2 completed
Jan 2003 to Dec 2005	2	Phases 1 & 2 completed	Phase 2 completed
Jan 2004 to Dec 2006	No observation	N.A.	N.A.
Jan 2005 to Dec 2007	3	Phases 1 & 2 & 3 completed	Phase 3 completed

As can be seen, our algorithm inferred all the phase transitions for the drug development project given the latest information at that point in time. While the algorithm works accurately when one has a massive database across long time horizons, it is unable to provide an accurate assessment of changes in success rates over short time windows. In our example, the Phase 1 trial is repeatedly counted as a success across multiple time windows, and this inflates the estimate of the success rate of Phase 1 trials in a short interval. When this situation occurs, we use the phase-by-phase approach.

A subtle but important difference between the two computation methods is that, while the path-by-path approach measures the proportion of *drug development projects* that progress, the phase-by-phase approach measures the proportion of *phase transitions* that occur. The two measures will produce the same results if there is no missing data point. However, these conditions do not hold true in real life clinical trial databases. By applying the phase-by-phase algorithm to the entire dataset, our evaluation is that it tends to underestimate the success rate. Nevertheless, the latter method is a strong enough proxy to estimate trends in drug development success rates.

A3. Algorithm

Algorithm 1 – Identifying trials in a drug development and computing the probability of success

```
Initialize count_12_succ = count_12_fail = count_23_succ = count_23_fail = count_3a_succ =
count_3a_fail = 0

for every pair {drug, indication}, do:

    Filter and populate a list of trials on indication using drug;

    if Drug is approved, then
        count_12_succ++;
        count_23_succ++;
        count_3a_succ++;
        continue;
    if there exists >=1 trial in Phase 3, then
        count_12_succ++;
        count_23_succ++;
        if latest end date of Phase 3 trials is < T – t3, then
            count_3a_fail++;
        continue;
    if there exists >=1 trial in Phase 2 then
        count_12_succ++;
        if latest end date of Phase 2 trials is < T – t2, then
            count_23_fail++;
        continue;
    if there exists >=1 trial in Phase 1 and if the latest end date is < T – t1, then
        count_12_fail++;
end
```

Fig. S5. An algorithm for identifying trials in drug development programs and computing the probability of success.

A4. All indications versus lead indications

The model and algorithm presented in SECTION A3 considered each drug-indication pair as a unique development path. Some analysts, however, are interested in the lead indication for a given drug, i.e., the indication that has progressed furthest in the development pipeline. If there is more than one indication in the highest phase of the pipeline, the indication that reached the phase first will be considered the lead indication. Indication B in Fig. S2 is the lead indication, as it is the only indication for which the drug is approved. We argue that using lead indications in financial analysis is problematic.

First, the definition of lead indication makes it confusing to analyze phase transition proba-

bilities. Consider the following example: Suppose that a company at time t completes Phase 2 clinical trials for two indications, Ind_A and Ind_B. It then decides to conduct a Phase 3 trial for Ind_A, making Ind_A the lead indication for the drug at $t+1$. A short time later, at $t+2$, the company reconsiders its priorities, and decides to accelerate development of the drug for Ind_B. Ind_B makes it to the market earlier than Ind_A, and is now the lead indication for the drug. Hence, depending on when one takes a snapshot of the data, one may end up with different lead indications and estimates of the indication-specific phase transition probabilities. As such, considering all indications in computing the phase transition probabilities is more robust and accurate.

Second, from a financial perspective, it may be more informative to use indication-specific drug development paths to compute the different metrics. Very often, a New Drug Application (NDA) specifies the indication and dosage that the drug is intended to treat, and a company would need to resubmit another application if they wish to market it for another disease or dosage. Since the patient segment determines the market size and thus the financial potential of the drug, it is more appropriate to use indication-specific probabilities in the financial analysis of drug development endeavors.

A5. Testing multiple indications

**Average Number of Indications Per Drug**					
	Mean	Std Dev		Mean	Std Dev
Oncology	2.61	3.24	Genitourinary	1.06	0.25
Metabolic/Endocrinology	1.38	0.71	Infectious Disease	1.5	0.72
Cardiovascular	1.3	0.65	Ophthalmology	1.25	0.48
CNS	1.26	0.60	Vaccines (Infectious Disease)	1.91	0.48
Autoimmune/Inflammation	1.34	0.81	Overall	1.74	1.95

Table S2. Average number of indications per drug, computed using the entire dataset from January 1, 2000, to October 31, 2015.

A6. Additional Results for Biomarker Trials

		Phase 1 to Phase 2			Phase 2 to Phase 3			Phase 3 to Approval			Overall	
**Therapeutic Group**		**Total Phase Transitions**	**$POS_{1,2}$, %**	**(SE, %)**	**Total Phase Transitions**	**$POS_{2,3}$, %**	**(SE, %)**	**Total Phase Transitions**	**$POS_{3,APP}$, %**	**(SE, %)**	**POS, %**	**(SE, %)**
Oncology	No Biomarker	5,499	26.3	(0.6)	3,190	16.2	(0.7)	903	33.6	(1.6)	1.4	(0.2)
	With Biomarker	4,986	33.5	(0.7)	2,325	25.8	(0.9)	333	40.8	(2.7)	3.5	(0.4)
	All	10,485	29.7	(0.4)	5,515	20.3	(0.5)	1,236	35.5	(1.4)	2.1	(0.2)
Metabolic/ Endocrinology	No Biomarker	1,424	45.5	(1.3)	1,214	34.5	(1.4)	865	54.1	(1.7)	8.5	(0.9)
	With Biomarker	115	33.0	(4.4)	226	31.0	(3.1)	236	42.4	(3.2)	4.3	(1.5)
	All	1,539	44.6	(1.3)	1,440	34.0	(1.2)	1,101	51.6	(1.5)	7.8	(0.8)
Cardiovascular	No Biomarker	1,117	38.1	(1.5)	711	36.8	(1.8)	673	67.5	(1.8)	9.5	(1.1)
	With Biomarker	131	55.0	(4.3)	321	41.1	(2.7)	291	50.2	(2.9)	11.3	(2.5)
	All	1,248	39.9	(1.4)	1,032	38.2	(1.5)	964	62.2	(1.6)	9.5	(1.0)
CNS	No Biomarker	2,011	40.3	(1.1)	1,858	29.9	(1.1)	1,049	51.2	(1.5)	6.2	(0.6)
	With Biomarker	212	43.9	(3.4)	234	32.5	(3.1)	107	50.5	(4.8)	7.2	(2.1)
	All	2,223	40.7	(1.0)	2,092	30.2	(1.0)	1,156	51.1	(1.5)	6.3	(0.6)
Autoimmune/ Inflammation	No Biomarker	2,227	37.7	(1.0)	1,765	24.9	(1.0)	867	64.0	(1.6)	6.0	(0.6)
	With Biomarker	288	49.0	(2.9)	355	28.5	(2.4)	102	60.8	(4.8)	8.5	(2.0)
	All	2,515	39.0	(1.0)	2,120	25.5	(0.9)	969	63.7	(1.5)	6.3	(0.6)
Genitourinary	No Biomarker	354	33.9	(2.5)	271	28.4	(2.7)	204	65.2	(3.3)	6.3	(1.5)
	With Biomarker	10	70.0	(14.5)	16	37.5	(12.1)	8	100.0	(0.0)	26.3	(15.7)
	All	364	34.9	(2.5)	287	28.9	(2.7)	212	66.5	(3.2)	6.7	(1.5)
Infectious Disease	No Biomarker	1,888	40.1	(1.1)	1,372	34.1	(1.3)	1,007	75.1	(1.4)	10.3	(0.9)
	With Biomarker	79	32.9	(5.3)	108	44.4	(4.8)	71	78.9	(4.8)	11.5	(4.2)
	All	1,967	39.8	(1.1)	1,480	34.9	(1.2)	1,078	75.3	(1.3)	10.5	(0.9)
Ophthalmology	No Biomarker	172	54.7	(3.8)	256	35.2	(3.0)	186	72.0	(3.3)	13.8	(3.0)
	With Biomarker	9	0.0	(0.0)	21	28.6	(9.9)	21	100.0	(0.0)	0.0	(0.0)
	All	181	51.9	(3.7)	277	34.7	(2.9)	207	74.9	(3.0)	13.5	(2.8)
Vaccines (Infectious Disease)	No Biomarker	718	41.4	(1.8)	748	33.2	(1.7)	597	85.8	(1.4)	11.8	(1.4)
	With Biomarker	15	13.3	(8.8)	18	11.1	(7.4)	12	66.7	(13.6)	1.0	(2.3)
	All	733	40.8	(1.8)	766	32.6	(1.7)	609	85.4	(1.4)	11.4	(1.3)
Overall	No Biomarker	15,410	35.3	(0.4)	11,385	27.0	(0.4)	6,351	60.7	(0.6)	5.8	(0.2)
	With Biomarker	5,845	35.0	(0.6)	3,624	28.8	(0.8)	1,181	50.0	(1.5)	5.0	(0.4)
	All	21,255	35.2	(0.3)	15,009	27.4	(0.4)	7,532	59.0	(0.6)	5.7	(0.2)

Table S3. Probability of success with and without biomarkers, using data from January 1, 2005, to October 31, 2015, computed using the phase-by-phase method. These results consider trials that have the objective of evaluating or identifying the use of any novel biomarkers as indicators of therapeutic efficacy or toxicity, in addition to patient stratification. Since for the majority (92.3%) of trials using biomarkers their status is observed only on or after January 1, 2005, the choice of the time period is to ensure a fair comparison between trials using and not using biomarkers.

A7. Comparison of results for biomarker trials against Thomas *and others* (2016)

Our results for trials using biomarkers are very different from extant papers such as Thomas *and others* (2016). The authors of Thomas *and others* (2016) kindly shared their analysis with us, allowing us to compare and contrast the methodologies and results. The main differences between the two analyses are in the identification of phase transitions, the application of filters, and the quantity of data (see Table S4).

	Thomas *and others* (2016)	This paper
Identification of phase transitions	From BioMedTracker database	Using Algorithm 1 in Figure S5
What constitutes a biomarker trial?	Considered only biomarkers in patient selection	Considered to 'involve biomarkers' if a trial includes includes an objective of evaluating or identifying the use of any novel biomarkers as indicators of therapeutic efficacy or toxicity, or to use biomarkers in the selection of patients.
Data source	Merges BioMedTracker with Amplions BiomarkerBase. Only trials from clinicaltrials.gov were used as NCT numbers were used as trial identifiers. Analysis consists of 512 phase transitions.	Uses trials tagged as 'involve biomarker' by Informa. Both clinicaltrials.gov and private information were used, summing up to 10,650 phase transitions.

Table S4. Differences between the biomarker study in Thomas *and others* (2016) and this paper.

Thomas *and others* (2016) provided a sample of 1,593 trial entries for comparison. Of these, 722 entries are used in their analysis. We merged our algorithm output with this subset of trials to produce tag outcomes for 1,065 of the 1,953 entries. Only 438 data points exist in both analyses. Our algorithm is unable to produce outcomes for some trials for which Thomas *and others* (2016) did because an insufficient period has passed since the conclusion of the trial. This relates to the *t1*, *t2*, and *t3* parameters in our algorithm.

Of the 438 overlapping data points, our algorithm arrived at the same conclusion as Thomas *and others* (2016) for 90.0% of the data, suggesting that our algorithm identifies phase transitions accurately.

Using this dataset of 1,065 identified entries, we compared our result against Thomas *and others* (2016) in Table S5. We see that our algorithm tends to identify more failures compared to Thomas *and others* (2016). This may be due to our method of counting a trial that is in limbo for an extended period of time as 'terminated'.

	Phase 1		Phase 2		Phase 3	
	Advanced	Terminated	Advanced	Terminated	Advanced	Terminated
Thomas *and others* (2016)	57	34	102	100	92	31
Our algorithm	37	23	172	170	164	102

Table S5. Comparison of identified phase transitions.

Given these checks, we conclude that our results differ from Thomas *and others* (2016) mainly due to the use of Algorithm 1 to process more trial data to produce POS estimates.

A8. PROBABILITY OF SUCCESS OVER TIME

The following tables supplement SECTION 4.4. We tabulate the POS over time for each therapeutic group.

Oncology										
Year	Phase 1			Phase 2			Phase 3			$POS_{1,APP}$
	Success	Failure	$POS_{1,2}$	Success	Failure	$POS_{2,3}$	Success	Failure	$POS_{3,APP}$	
2005	812	1297	38.5%	410	771	34.7%	155	176	46.8%	6.3%
2006	946	1410	40.2%	486	909	34.8%	144	212	40.4%	5.7%
2007	1014	1368	42.6%	496	1022	32.7%	142	241	37.1%	5.2%
2008	1005	1419	41.5%	509	1112	31.4%	142	269	34.5%	4.5%
2009	1026	1640	38.5%	490	1237	28.4%	145	270	34.9%	3.8%
2010	1083	1942	35.8%	511	1369	27.2%	139	291	32.3%	3.1%
2011	1098	2344	31.9%	488	1516	24.4%	120	251	32.3%	2.5%
2012	1091	2739	28.5%	481	1752	21.5%	116	298	28.0%	1.7%
2013	1067	2830	27.4%	449	1843	19.6%	131	248	34.6%	1.9%
2014	1006	2727	26.9%	423	1505	21.9%	139	193	41.9%	2.5%
2015	862	1733	33.2%	399	843	32.1%	118	33	78.1%	8.3%

Table S6. POS for oncology trials between the years 2005 and 2015, computed using a rolling window of 3 years.

Metabolic/ Endocrinology										
Year	Phase 1			Phase 2			Phase 3			$POS_{1,APP}$
	Success	Failure	$POS_{1,2}$	Success	Failure	$POS_{2,3}$	Success	Failure	$POS_{3,APP}$	
2005	154	65	70.3%	206	167	55.2%	142	168	45.8%	17.8%
2006	204	85	70.6%	207	208	49.9%	164	168	49.4%	17.4%
2007	231	146	61.3%	180	233	43.6%	179	187	48.9%	13.1%
2008	257	216	54.3%	183	283	39.3%	171	219	43.8%	9.4%
2009	241	262	47.9%	171	305	35.9%	159	227	41.2%	7.1%
2010	270	324	45.5%	178	365	32.8%	171	208	45.1%	6.7%
2011	266	332	44.5%	173	363	32.3%	172	188	47.8%	6.9%
2012	275	339	44.8%	173	358	32.6%	179	181	49.7%	7.3%
2013	240	346	41.0%	144	298	32.6%	177	136	56.5%	7.5%
2014	213	306	41.0%	134	223	37.5%	208	92	69.3%	10.7%
2015	193	201	49.0%	105	115	47.7%	179	13	93.2%	21.8%

Table S7. POS for metabolic/endocrinology trials between the years 2005 and 2015, computed using a rolling window of 3 years.

Cardiovascular										
Year	Phase 1			Phase 2			Phase 3			$POS_{1,APP}$
	Success	Failure	$POS_{1,2}$	Success	Failure	$POS_{2,3}$	Success	Failure	$POS_{3,APP}$	
2005	113	87	56.5%	147	129	53.3%	168	93	64.4%	19.4%
2006	143	105	57.7%	151	139	52.1%	167	116	59.0%	17.7%
2007	171	148	53.6%	145	157	48.0%	170	143	54.3%	14.0%
2008	191	173	52.5%	129	180	41.7%	188	146	56.3%	12.3%
2009	199	208	48.9%	124	188	39.7%	178	145	55.1%	10.7%
2010	192	222	46.4%	131	229	36.4%	187	130	59.0%	10.0%
2011	198	251	44.1%	139	244	36.3%	151	139	52.1%	8.3%
2012	178	257	40.9%	129	236	35.3%	166	138	54.6%	7.9%
2013	163	292	35.8%	120	195	38.1%	152	106	58.9%	8.0%
2014	140	266	34.5%	93	125	42.7%	191	65	74.6%	11.0%
2015	122	174	41.2%	88	63	58.3%	189	10	95.0%	22.8%

Table S8. POS for cardiovascular trials between the years 2005 and 2015, computed using a rolling window of 3 years.

CNS										
Year	Phase 1			Phase 2			Phase 3			$POS_{1,APP}$
	Success	Failure	$POS_{1,2}$	Success	Failure	$POS_{2,3}$	Success	Failure	$POS_{3,APP}$	
2005	191	107	64.1%	245	269	47.7%	170	164	50.9%	15.5%
2006	235	146	61.7%	269	331	44.8%	194	177	52.3%	14.5%
2007	252	208	54.8%	254	363	41.2%	222	222	50.0%	11.3%
2008	282	286	49.6%	233	439	34.7%	218	241	47.5%	8.2%
2009	344	439	43.9%	211	451	31.9%	228	249	47.8%	6.7%
2010	400	537	42.7%	215	480	30.9%	225	236	48.8%	6.4%
2011	385	579	39.9%	206	468	30.6%	217	225	49.1%	6.0%
2012	345	546	38.7%	186	456	29.0%	219	207	51.4%	5.8%
2013	307	498	38.1%	177	455	28.0%	225	175	56.3%	6.0%
2014	293	439	40.0%	184	362	33.7%	207	108	65.7%	8.9%
2015	238	281	45.9%	146	228	39.0%	178	18	90.8%	16.3%

Table S9. POS for CNS trials between the years 2005 and 2015, computed using a rolling window of 3 years.

Autoimmune/ Inflammation										
Year	Phase 1			Phase 2			Phase 3			$POS_{1,APP}$
	Success	Failure	$POS_{1,2}$	Success	Failure	$POS_{2,3}$	Success	Failure	$POS_{3,APP}$	
2005	208	169	55.2%	188	350	34.9%	198	104	65.6%	12.6%
2006	246	193	56.0%	191	388	33.0%	200	117	63.1%	11.7%
2007	267	233	53.4%	177	400	30.7%	206	118	63.6%	10.4%
2008	296	274	51.9%	166	444	27.2%	213	126	62.8%	8.9%
2009	301	362	45.4%	186	471	28.3%	227	147	60.7%	7.8%
2010	310	487	38.9%	183	500	26.8%	227	159	58.8%	6.1%
2011	316	544	36.7%	184	490	27.3%	202	150	57.4%	5.8%
2012	299	612	32.8%	191	489	28.1%	211	156	57.5%	5.3%
2013	292	600	32.7%	186	466	28.5%	201	121	62.4%	5.8%
2014	289	580	33.3%	172	387	30.8%	189	76	71.3%	7.3%
2015	250	354	41.4%	142	212	40.1%	158	19	89.3%	14.8%

Table S10. POS for autoimmune/inflammation trials between the years 2005 and 2015, computed using a rolling window of 3 years.

Genitourinary										
Year	Phase 1			Phase 2			Phase 3			$POS_{1,APP}$
	Success	Failure	$POS_{1,2}$	Success	Failure	$POS_{2,3}$	Success	Failure	$POS_{3,APP}$	
2005	25	26	49.0%	34	35	49.3%	32	11	74.4%	18.0%
2006	30	41	42.3%	39	48	44.8%	51	18	73.9%	14.0%
2007	46	67	40.7%	35	52	40.2%	53	25	67.9%	11.1%
2008	46	89	34.1%	36	68	34.6%	59	33	64.1%	7.6%
2009	56	86	39.4%	32	73	30.5%	60	26	69.8%	8.4%
2010	45	78	36.6%	31	81	27.7%	63	26	70.8%	7.2%
2011	47	77	37.9%	23	67	25.6%	57	26	68.7%	6.7%
2012	40	77	34.2%	21	55	27.6%	51	30	63.0%	5.9%
2013	37	68	35.2%	25	43	36.8%	41	24	63.1%	8.2%
2014	27	68	28.4%	22	44	33.3%	35	13	72.9%	6.9%
2015	31	47	39.7%	18	34	34.6%	33	3	91.7%	12.6%

Table S11. POS for genitourinary trials between the years 2005 and 2015, computed using a rolling window of 3 years.

Infectious Disease										
Year	Phase 1			Phase 2			Phase 3			$POS_{1,APP}$
	Success	Failure	$POS_{1,2}$	Success	Failure	$POS_{2,3}$	Success	Failure	$POS_{3,APP}$	
2005	134	124	51.9%	170	191	47.1%	159	97	62.1%	15.2%
2006	170	137	55.4%	170	195	46.6%	201	110	64.6%	16.7%
2007	212	166	56.1%	189	215	46.8%	252	88	74.1%	19.4%
2008	234	185	55.8%	188	249	43.0%	291	96	75.2%	18.1%
2009	253	284	47.1%	194	309	38.6%	347	115	75.1%	13.6%
2010	239	355	40.2%	185	352	34.5%	343	109	75.9%	10.5%
2011	258	454	36.2%	197	349	36.1%	332	81	80.4%	10.5%
2012	287	497	36.6%	187	368	33.7%	299	83	78.3%	9.7%
2013	314	475	39.8%	154	344	30.9%	283	68	80.6%	9.9%
2014	326	472	40.9%	140	265	34.6%	276	42	86.8%	12.3%
2015	282	312	47.5%	113	153	42.5%	230	7	97.0%	19.6%

Table S12. POS for infectious disease trials between the years 2005 and 2015, computed using a rolling window of 3 years.

Ophthalmology										
Year	Phase 1			Phase 2			Phase 3			$POS_{1,APP}$
	Success	Failure	$POS_{1,2}$	Success	Failure	$POS_{2,3}$	Success	Failure	$POS_{3,APP}$	
2005	7	5	58.3%	21	25	45.7%	28	13	68.3%	18.2%
2006	13	8	61.9%	28	30	48.3%	37	16	69.8%	20.9%
2007	20	16	55.6%	31	29	51.7%	33	17	66.0%	18.9%
2008	26	27	49.1%	35	39	47.3%	29	25	53.7%	12.5%
2009	31	36	46.3%	36	53	40.4%	38	23	62.3%	11.7%
2010	32	28	53.3%	42	69	37.8%	48	31	60.8%	12.3%
2011	29	21	58.0%	45	82	35.4%	49	28	63.6%	13.1%
2012	36	22	62.1%	46	78	37.1%	41	26	61.2%	14.1%
2013	40	34	54.1%	43	68	38.7%	44	11	80.0%	16.8%
2014	38	32	54.3%	41	53	43.6%	75	3	96.2%	22.8%
2015	26	21	55.3%	33	28	54.1%	76	1	98.7%	29.5%

Table S13. POS for ophthalmology trials between the years 2005 and 2015, computed using a rolling window of 3 years.

Vaccines (Infectious Disease)										
Year	Phase 1			Phase 2			Phase 3			$POS_{1,APP}$
	Success	Failure	$POS_{1,2}$	Success	Failure	$POS_{2,3}$	Success	Failure	$POS_{3,APP}$	
2005	23	58	28.4%	71	89	44.4%	80	30	72.7%	9.2%
2006	43	63	40.6%	85	88	49.1%	116	38	75.3%	15.0%
2007	69	73	48.6%	116	107	52.0%	172	31	84.7%	21.4%
2008	90	91	49.7%	111	134	45.3%	217	34	86.5%	19.5%
2009	106	114	48.2%	106	180	37.1%	239	31	88.5%	15.8%
2010	93	116	44.5%	103	216	32.3%	248	32	88.6%	12.7%
2011	95	120	44.2%	111	210	34.6%	236	30	88.7%	13.6%
2012	100	145	40.8%	99	205	32.6%	239	31	88.5%	11.8%
2013	97	172	36.1%	72	190	27.5%	203	29	87.5%	8.7%
2014	98	171	36.4%	63	148	29.9%	187	17	91.7%	10.0%
2015	78	110	41.5%	44	92	32.4%	157	2	98.7%	13.3%

Table S14. POS for Vaccines (Infectious Disease) trials between the years 2005 and 2015, computed using a rolling window of 3 years.

A9. Trials per development path

In this section, we record the average number of trials per development path. From Table S15, we see that the average number of Phase 1, Phase 2, Phase 3, and Phase 4 trials for a drug development are 1.7, 2.0, 2.8, and 3.2, respectively. The high number of Phase 4 trials per development path is surprising, as it indicates that many approved drugs require substantial long-term studies to identify and evaluate long-term side effects.

	Phase 1	Phase 2	Phase 3	Phase 4
Oncology	1.6	2.4	2.3	2.2
Metabolic/ Endocrinology	2.2	1.9	3.1	3.2
Cardiovascular	2.0	1.9	2.9	3.4
CNS	2.0	1.7	3.2	3.4
Autoimmune/ Inflammation	1.6	1.6	3.2	3.4
Genitourinary	1.7	1.7	2.4	2.6
Infectious Disease	2.0	1.9	2.9	3.1
Ophthalmology	1.2	1.8	2.2	3.9
Vaccines (Infectious Disease)	1.3	2.0	3.0	2.8
Overall	1.7	2.0	2.8	3.2

Table S15. Average number of trials per development path, computed for all indications over the period of January 1, 2000, to October 31, 2015.

A10. Completion Rates

An alternative measure of performance for clinical trials is the completion rate. It answers the question, “How likely is a trial to complete?” The completion rate of Phase i trials (CR_i) is computed by dividing the number of trials in Phase i that were tagged as ‘completed’ by the number of trials that have been initiated in Phase i. This metric is useful in real option valuation, where uncertain possible outcomes with various endpoints are implicitly modeled in order to provide a more robust and comprehensive cost-benefit analysis. Our data shows that clinical trial completion rates are high across all phases, averaging at 85.8% (Table S16). Phase 2 trials have the lowest tendency to complete, with only 81.1% of all trials being completed. On the other hand, 91.3% of all Phase 1 trials are completed. While Phase 3 trials are often larger-scale replications

of Phase 2 trials, and thus potentially riskier and costlier, they have a higher completion rate than Phase 2 trials. Possible explanations include selection bias and commitment, as only the most promising trials in Phase 2 are selected for Phase 3 trials and given sufficient resources to complete the trials since they are paramount in getting marketing approval.

Differences emerge after breaking down the completion rates of clinical trials by therapeutic group. With the exception of cancer-treating drugs, most drug development projects have a trial completion rate between 84.4% and 93.1%. Oncology trials performed much more poorly than average, with only 73.9% of all trials concluding successfully. A closer look shows that their completion rates were lower across all phases, pointing to a possible bottleneck in the development of oncology drugs.

The completion rates for non-industry sponsored trials are provided in SECTION A13.

	Phase 1			Phase 2			Phase 3			Phase 4		
	Completed	Failed	CR_1	Completed	Failed	CR_2	Completed	Failed	CR_3	Completed	Failed	CR_4
Oncology	3910	885	81.5%	6278	2501	71.5%	1439	706	67.1%	403	149	73.0%
Metabolic/ Endocrinology	2602	145	94.7%	1939	292	86.9%	2267	370	86.0%	1564	227	87.3%
Cardiovascular	1884	110	94.5%	1349	249	84.4%	1679	290	85.3%	1373	199	87.3%
CNS	3233	185	94.6%	2862	432	86.9%	3091	453	87.2%	2100	245	89.6%
Autoimmune/ Inflammation	2449	132	94.9%	2986	432	87.4%	2681	343	88.7%	1984	234	89.4%
Genitourinary	507	16	96.9%	419	56	88.2%	450	53	89.5%	324	43	88.3%
Infectious Disease	2424	140	94.5%	1715	268	86.5%	1698	243	87.5%	1111	220	83.5%
Ophthalmology	161	18	89.9%	424	72	85.5%	307	51	85.8%	336	45	88.2%
Vaccines (Infectious Disease)	414	37	91.8%	752	69	91.6%	850	63	93.1%	337	34	90.8%
Total	17584	1668	91.3%	18724	4371	81.1%	14462	2572	84.9%	9532	1396	87.2%

Table S16. Completion rates of industry-sponsored clinical trials (i.e., the number of trials that were tagged as completed divided by the number of trials that were initiated) by phase and therapeutic group, using the entire dataset from January 1, 2000, to October 31, 2015.

A11. Duration

One principal component of the cost of conducting a trial is its expected duration. All else being equal, one would expect that a longer trial would require more hours of labor and supplies, resulting in a higher cost. In addition, from a financial perspective, a longer trial is exposed to more uncertainties. We quantify the distribution of the duration of trials in order to inform companies and investors of the potential risk in a project. We assume that there is no underlying process that induces gaps in the data. We drop trial data without date-stamps for the start or the end of the trial, as we cannot make a statement on the time spent in development for these trials. After data processing, 99,363 trials remain for our computations. Our data has a resolution of 1 calendar month.

The distribution of duration varies widely across different therapeutic groups and phases (Table S17). A typical trial takes a median time of 1.61, 2.94, and 3.84 years to complete Phase 1, Phase 2, and Phase 3, respectively. Simply by summing up the median time in Phase 1, 2, and 3, we approximate that the typical drug spends an average of 8.14 years in clinical trials. This number excludes the preparation time and other factors which may lengthen the overall clinical trial cycle time. While the median duration for other therapeutic groups lies between 5.94 to 7.15 years, oncology trials take 13.11 years. This causes higher risks in oncology projects, and may explain their low approval rate. The empirical distributions and the gamma-kernelled non-parametric density estimates (see Malec and Schienle (2014) for computation details) are plotted in the following section.

Taking cues from Abrantes-Metz *and others* (2005), we also compute the duration of trials conditioned on their eventual status ('advanced' or 'terminated') using a 5-year rolling window (Figure S6). With our larger dataset, we found that Phase 2 trials that were terminated tend to conclude 8.1 months earlier than Phase 2 trials that advanced (Table S18). Terminated Phase 3 trials, however, tend to conclude about 3.2 months after Phase 3 trials that successfully advanced.

	Phase 1	Phase 2	Phase 3	Phase 4
Oncology	1216	1490	2080	1394
Metabolic/ Endocrinology	325	946	976	1036
Cardiovascular	379	1025	1208	1174
CNS	334	932	1034	1068
Autoimmune/ Inflammation	335	980	979	1207
Genitourinary	378	787	1005	913
Infectious Disease	562	951	1067	1180
Ophthalmology	546	823	1028	935
Vaccines (Infectious Disease)	714	827	798	900

Table S17. Median duration of trials in days. Only data entries with date stamps from January 1, 2000, to October 31, 2015 are used.

The difference within the Phase 1 group is insignificant; while we see a difference of 53 days, this is within our margin of error, given that the resolution for a time period is 2 calendar months, or 60 days. By composing a time series using 5-year rolling windows (see Figure S6), we see that the differences (or lack thereof) remain constant over time.

	Terminated	Advanced	Difference ('Advanced' - 'Terminated')
Phase 1	487	540	53.0
Phase 2	823	1065	242.0
Phase 3	1035	941	-94.0

Table S18. Median duration of trials conditioned on eventual status, in days.

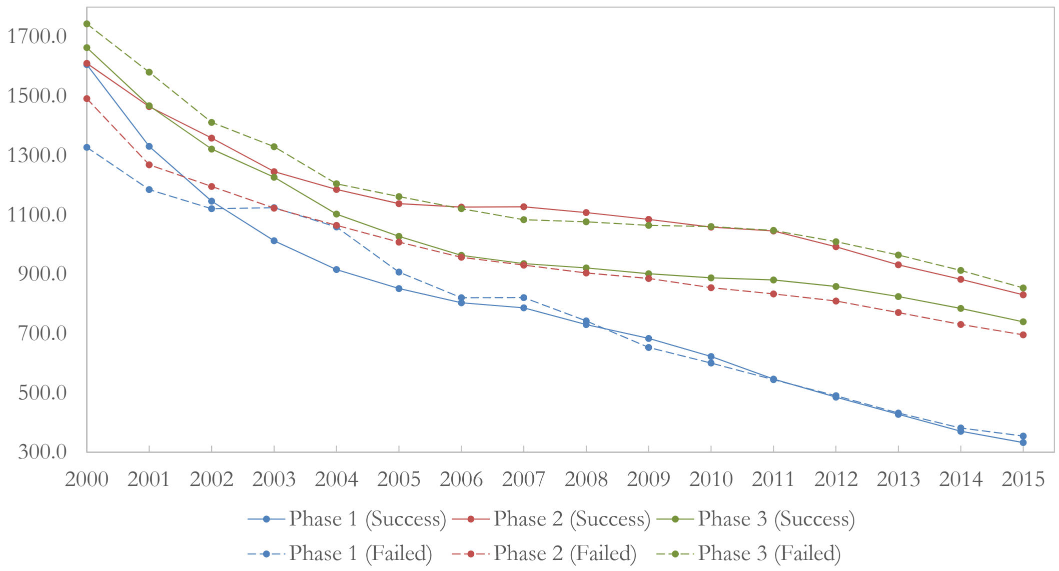

Fig. S6. Plot of median duration of trials across time.

A12. Distribution of Duration

In this section, we document the distribution of duration conditioned on the indication group and phase in order to inform interested readers.

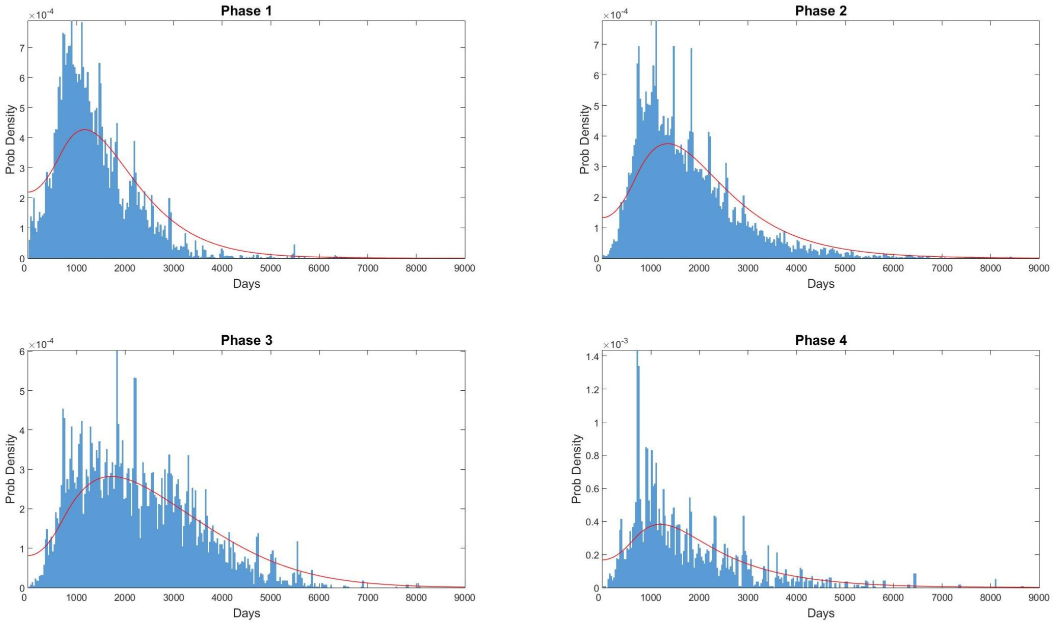

Fig. S7. Distribution of duration for oncology trials conditioned on the phase.

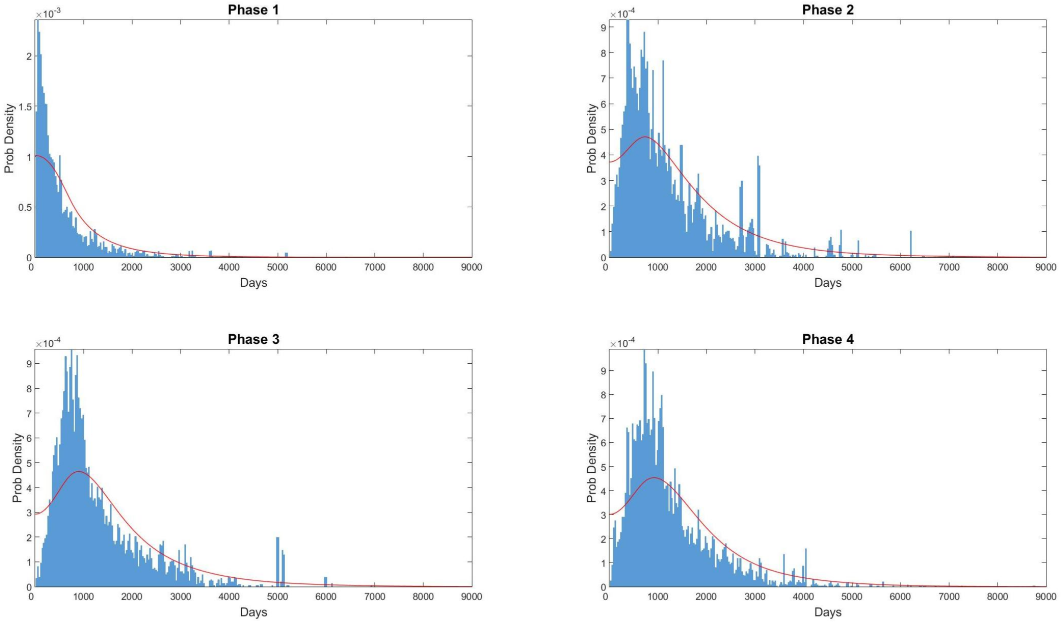

Fig. S8. Distribution of duration for metabolic/endocrinology trials conditioned on the phase.

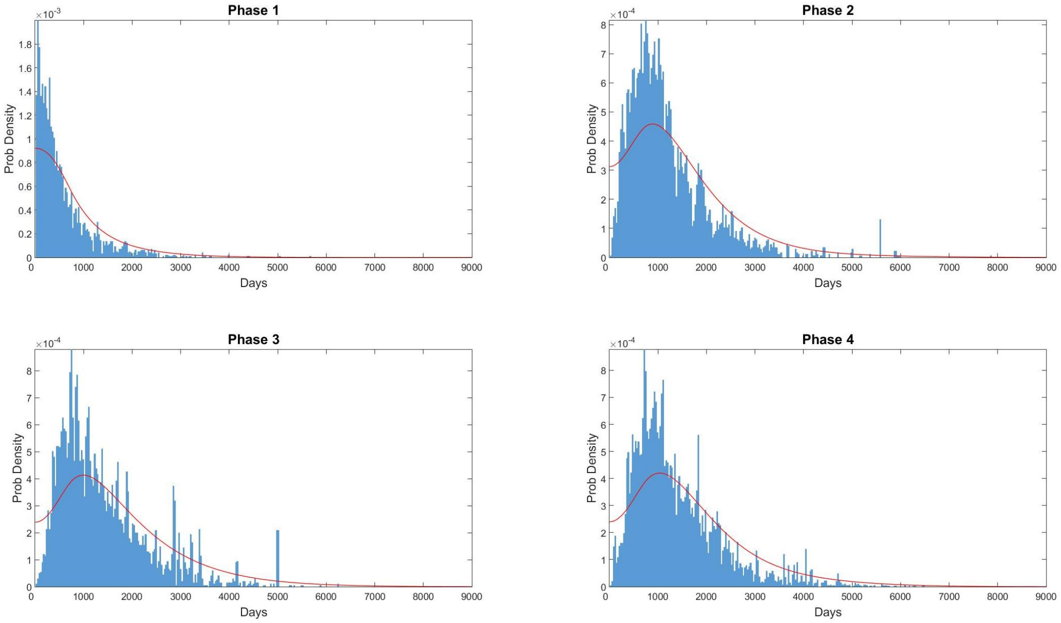

Fig. S9. Distribution of duration for cardiovascular trials conditioned on the phase.

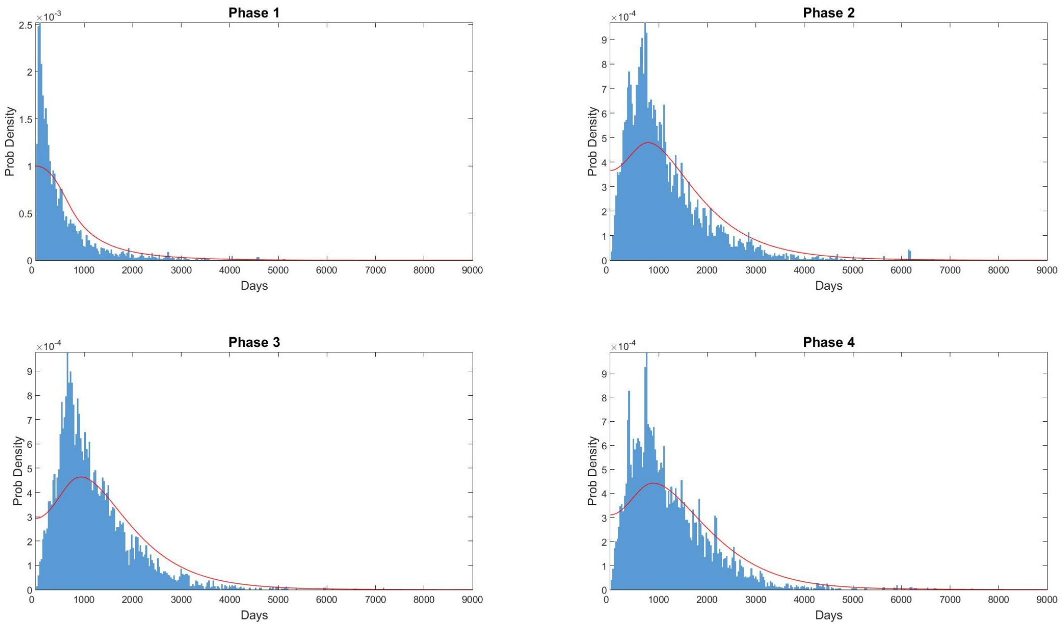

Fig. S10. Distribution of duration for CNS trials conditioned on the phase.

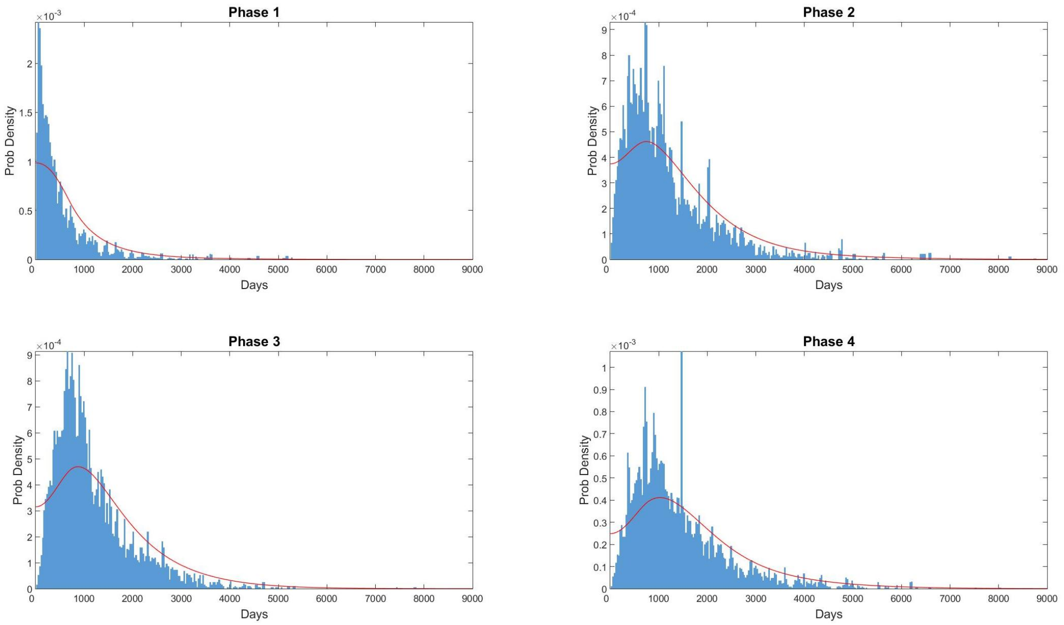

Fig. S11. Distribution of duration for autoimmune/inflammation trials conditioned on the phase.

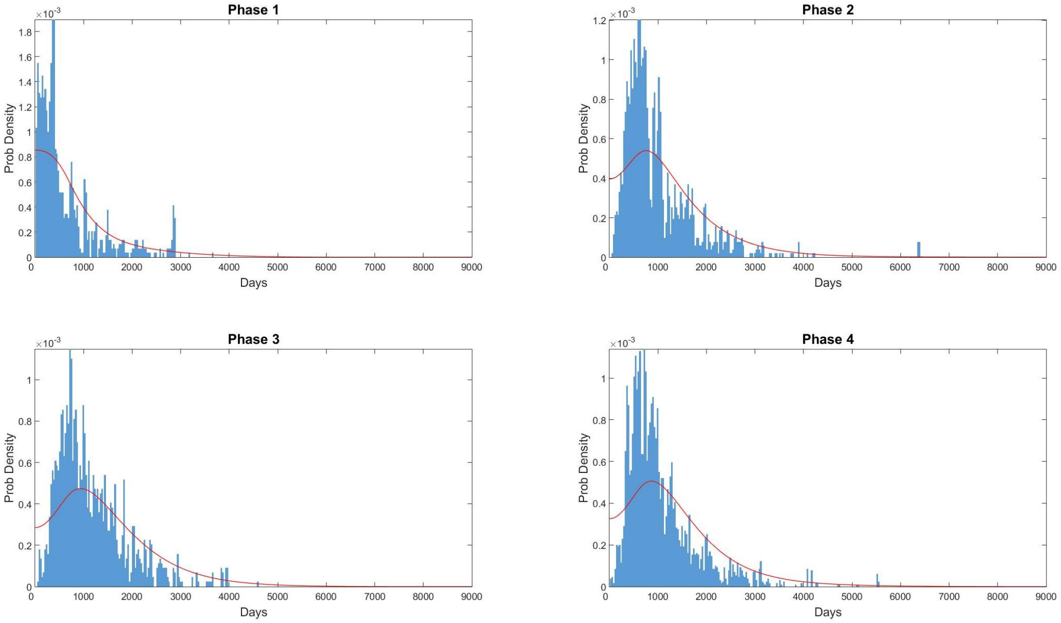

Fig. S12. Distribution of duration for genitourinary trials conditioned on the phase.

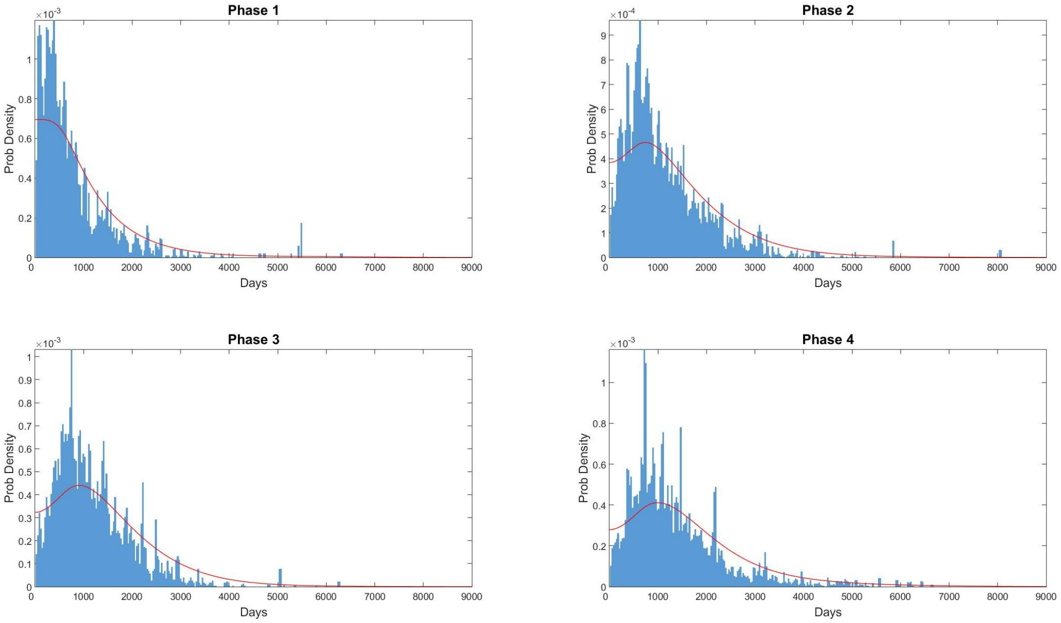

Fig. S13. Distribution of duration for infectious disease trials conditioned on the phase.

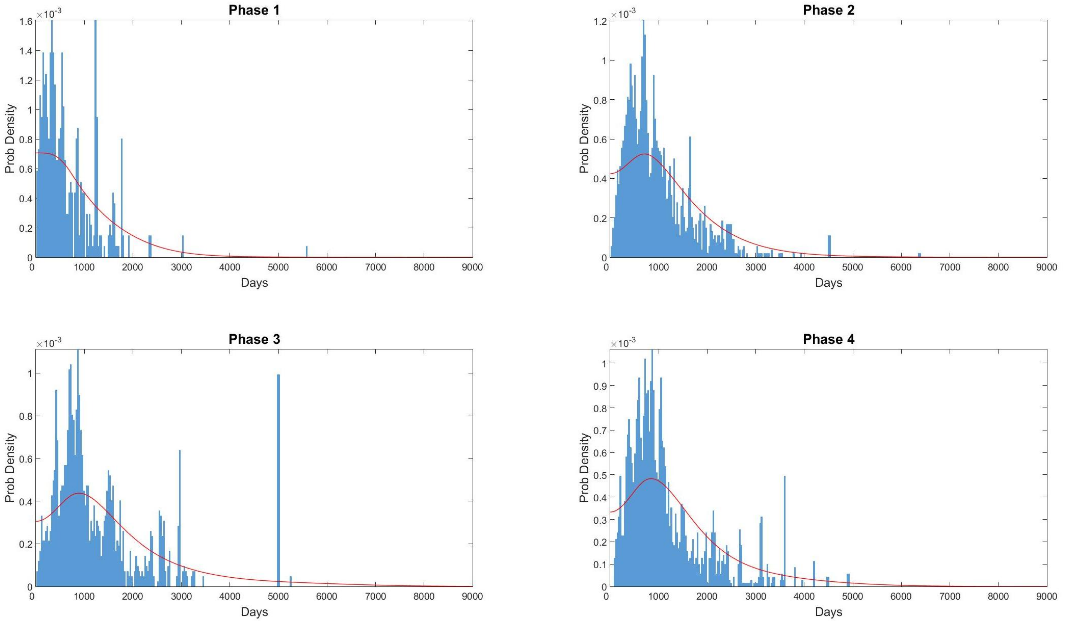

Fig. S14. Distribution of duration for ophthalmology trials conditioned on the phase.

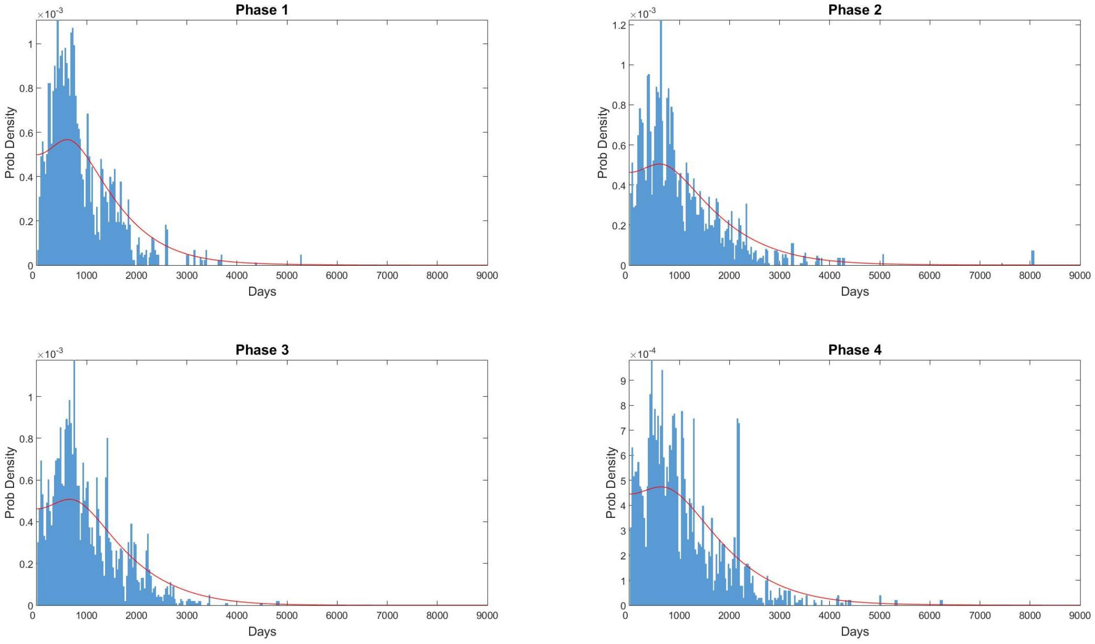

Fig. S15. Distribution of duration for vaccines (infectious disease) trials conditioned on the phase.

A13. Non-Industry Trials

The clinical research sector outside the pharmaceutical industry is an integral part of drug research and development. Not only is this sector actively involved with industry in conducting trials, but academics and hospitals also conduct fundamental research that furthers understanding of basic pharmacokinetics, among other phenomena measured in clinical trials. We thus seek to quantify the performance of this sector.

As our database does not record non-industry approvals, we supplement our dataset with data from *Drugs@FDA*, the U.S. Food and Drug Administration's (FDA) approved drugs database. In all, 53 drug approvals for 17 unique compounds were awarded to non-industry organizations (see Table S20). Of these, only three compounds were non-generic: two were awarded to the U.S. Army and the remaining compound is a PET imaging diagnostic agent. The remaining drugs are generic compounds whose patents have expired and have been awarded to hospitals and non-profits.

Given the altruistic aims of organizations outside the industry, and the fact that virtually no novel drugs have been granted by the FDA to these organizations, we look at only the completion rates for non-industry trials. We find that, although Phase 1 trials conducted outside the industry have lower completion rates than those within the industry, non-industry organizations outperform the latter in completing Phase 2, Phase 3, and Phase 4 trials (compare Tables S16 and S19). This suggests that each group has a relative advantage in completing different phases of clinical trials, and that there may be exploitable synergies to be gained when working together. Computing the POS of drug development projects conditioned on the status and number of non-industry partners (Table S21) shows that drug development projects involving non-industry partners have a 5% higher chance of getting marketing approval for their drugs. These results extend the findings by Danzon *and others* (2005).

	Phase 1			Phase 2			Phase 3			Phase 4		
	Completed	Failed	CR_1	Completed	Failed	CR_2	Completed	Failed	CR_3	Completed	Failed	CR_4
Oncology	2,327	511	82.0%	12,199	2474	83.1%	1,379	527	72.4%	592	83	87.7%
Metabolic/ Endocrinology	323	26	92.6%	2,351	157	93.7%	1,073	134	88.9%	5,446	280	95.1%
Cardiovascular	461	32	93.5%	4,676	178	96.3%	1,340	144	90.3%	7,106	318	95.7%
CNS	564	60	90.4%	5,677	404	93.4%	2,068	257	88.9%	7,507	537	93.3%
Autoimmune/ Inflammation	431	37	92.1%	4,046	236	94.5%	1,589	105	93.8%	6,156	210	96.7%
Genitourinary	84	9	90.3%	918	45	95.3%	334	47	87.7%	1,741	126	93.3%
Infectious Disease	702	76	90.2%	2,264	220	91.1%	1,030	146	87.6%	4,887	374	92.9%
Ophthalmology	60	7	89.6%	1,238	28	97.8%	361	22	94.3%	1,642	50	97.0%
Vaccines (Infectious Disease)	335	60	84.8%	450	50	90.0%	192	15	92.8%	807	73	91.7%
Total	5,287	818	86.6%	33,819	3792	89.9%	9,366	1397	87.0%	35,884	2,051	94.6%

Table S19. Completion rates of non-industry-sponsored trials based on data from January 1, 2000, to October 31, 2015.

Generic?	SponsorName	ApplNo	Drugname
No	BIOMEDCL RES FDN	204352	AMMONIA N 13
No	BIOMEDCL RES FDN	203710	FLUDEOXYGLUCOSE F18
Yes	BIOMEDCL RES FDN	204351	SODIUM FLUORIDE F-18
No	BRIGHAM WOMENS	203816	FLUDEOXYGLUCOSE F18
No	BRIGHAM WOMENS HOSP	203783	AMMONIA N 13
No	CHILDRENS HOSP MI	204385	FLUDEOXYGLUCOSE F18
No	FEINSTEIN	22119	AMMONIA N 13
No	FEINSTEIN	21870	FLUDEOXYGLUCOSE F18
No	FEINSTEIN	21870	FLUDEOXYGLUCOSE F18
No	HEALTHPOINT	84698	NUTRACORT
No	HOUSTON CYCLOTRON	203543	AMMONIA N 13
No	HOUSTON CYCLOTRON	203665	FLUDEOXYGLUCOSE F18
Yes	HOUSTON CYCLOTRON	203544	SODIUM FLUORIDE F-18
No	JOHNS HOPKINS UNIV	204514	AMMONIA N 13
No	KETTERING MEDCTR	204759	FLUDEOXYGLUCOSE F18
No	KREITCHMAN PET CTR	203938	AMMONIA N 13
No	KREITCHMAN PET CTR	203942	FLUDEOXYGLUCOSE F18
Yes	KREITCHMAN PET CTR	203936	SODIUM FLUORIDE F-18
No	MA GENERAL HOSP	207025	AMMONIA N 13
No	MA GENERAL HOSP	204333	FLUDEOXYGLUCOSE F18
No	METHODIST HOSP RES	203904	FLUDEOXYGLUCOSE F18
No	NIH NCI DCTD	22494	SODIUM FLUORIDE F 18
No	POPULATION COUNCIL	20544	JADELLE
No	POPULATION COUNCIL	19897	NORPLANT
No	QUEEN HAMAMATSU PET	203771	FLUDEOXYGLUCOSE F18
Yes	THE FEINSTEIN INST	204328	SODIUM FLUORIDE F-18
No	TRUSTEES UNIV PA	203801	FLUDEOXYGLUCOSE F18
No	UCLA BIOMEDICAL	203812	AMMONIA N 13
No	UCLA BIOMEDICAL	203811	FLUDEOXYGLUCOSE F18
No	UIHC PET IMAGING	203990	FLUDEOXYGLUCOSE F18
Yes	UIHC PET IMAGING	204462	SODIUM FLUORIDE F-18
No	UNIV AZ CANCER CTR	19940	ACTINEX
No	UNIV MICHIGAN	204531	FLUDEOXYGLUCOSE F18
No	UNIV NORTH DAKOTA	203994	FLUDEOXYGLUCOSE F18
No	UNIV TX MD ANDERSON	203933	AMMONIA N 13
No	UNIV TX MD ANDERSON	205690	CHOLINE C-11
No	UNIV TX MD ANDERSON	203246	FLUDEOXYGLUCOSE F18
Yes	UNIV TX MD ANDERSON	203247	SODIUM FLUORIDE F-18
No	UNIV UTAH CYCLOTRON	204498	FLUDEOXYGLUCOSE F18
Yes	UNIV UTAH CYCLOTRON	204497	SODIUM FLUORIDE F-18
No	US ARMY	21175	ATNAA
Yes	US ARMY	20056	ATROPINE SULFATE
No	US ARMY	20124	DIAZEPAM
Yes	US ARMY	20414	PYRIDOSTIGMINE BROMIDE
No	US ARMY	21084	SKIN EXPOSURE REDUCTION PASTE AGAINST CHEMICAL WARFARE AGENTS
No	US ARMY	20166	SODIUM THIOSULFATE
No	US ARMY WALTER REED	19578	MEFLOQUINE HYDROCHLORIDE
No	UT SW MEDCTR	19647	POTASSIUM CITRATE
No	WA UNIV SCH MED	204506	AMMONIA N 13
No	WEILL MEDCL COLL	21768	FLUDEOXYGLUCOSE F18
No	WI MEDCL CYCLOTRON	204356	AMMONIA N 13
No	WI MEDCL CYCLOTRON	203709	FLUDEOXYGLUCOSE F18
No	WUSM CYCLOTRON	203935	FLUDEOXYGLUCOSE F18
No	BIOMEDCL RES FDN	203837	FLUDEOXYGLUCOSE F18
No	UNIV TX MD ANDERSON	203246	FLUDEOXYGLUCOSE F18
No	UT SW MEDCTR	19647	POTASSIUM CITRATE

Table S20. Table of drug approvals awarded to non-industry organizations, extracted from Drugs@FDA.

A14. Success rates of trials with non-industry partners

Overall			
Number of non-industry partner(s)	Advanced	Failed or Terminated	POS
0	9631	10250	48.4%
1	11338	8328	57.7%
2	3645	2290	61.4%
3	986	398	71.2%
4	320	106	75.1%
5	137	35	79.7%
6	73	7	91.3%
>6	65	17	79.3%
Joint (>0 partners)	16564	11181	59.7%

Table S21. Overall success rates of trials with non-industry partners, based on data from January 1, 2000, to October 31, 2015.

Phase 1			
Number of non-industry partner(s)	Advanced	Failed or Terminated	POS
0	4235	4207	50.2%
1	2350	1444	61.9%
2	918	592	60.8%
3	173	100	63.4%
4	40	15	72.7%
5	9	4	69.2%
6	8	2	80.0%
>6	1	0	100.0%
Joint (>0 partners)	3499	2157	61.9%

Table S22. Phase 1 success rates of trials with non-industry partners, based on data from January 1, 2000, to October 31, 2015.

Phase 2			
Number of non-industry partner(s)	Advanced	Failed or Terminated	POS
0	3063	4779	39.1%
1	5314	5953	47.2%
2	1667	1418	54.0%
3	459	241	65.6%
4	157	64	71.0%
5	55	22	71.4%
6	22	3	88.0%
>6	19	11	63.3%
Joint (>0 partners)	7693	7712	49.9%

Table S23. Phase 2 success rates of trials with non-industry partners, based on data from January 1, 2000, to October 31, 2015.

Phase 3			
Number of non-industry partner(s)	Advanced	Failed or Terminated	POS
0	2333	1264	64.9%
1	3674	931	79.8%
2	1060	280	79.1%
3	354	57	86.1%
4	123	27	82.0%
5	73	9	89.0%
6	43	2	95.6%
>6	45	6	88.2%
Joint (>0 partners)	5372	1312	80.4%

Table S24. Phase 3 success rates of trials with non-industry partners, based on data from January 1, 2000, to October 31, 2015.

A15. Robustness Checks

These tables supplement SECTION 5.

Comparison of $POS_{1,APP}$ on various subsets of the data			
	All data (%)	2006-2015 (%)	ClinicalTrials.gov only (%)
Oncology	3.4	2.9	2.6
Metabolic/ Endocrinology	19.6	17.5	19.2
Cardiovascular	25.5	23.8	26.6
CNS	15.0	13.6	15.1
Autoimmune/ Inflammation	15.1	13.9	14.6
Genitourinary	21.6	21.0	24.4
Infectious Disease	25.2	25.6	27.2
Ophthalmology	32.6	31.3	34.8
Vaccines (Infectious Disease)	33.4	34.8	35.5
Overall	13.8	13.2	13.4

Table S25. Robustness checks: comparison of various subsets of the data against the entire dataset.

Trials occurring between 2006-2015								
	Phase 1 to Phase 2		Phase 2 to Phase 3			Phase 3 to Approval		Overall
Therapeutic Group	Total Paths	$POS_{1,2}$, %	Total Paths	$POS_{2,3}$, %	$POS_{2,APP}$, %	Total Paths	$POS_{3,APP}$, %	$POS_{1,APP}$, %
Oncology	15,192	59.8	5,616	23.1	33.2	6,355	33.1	2.9
Metabolic/ Endocrinology	3,173	74.7	1,989	58.4	58.9	2,719	50.6	17.5
Cardiovascular	2,400	72.8	1,543	72.1	66.6	3,380	60.3	23.8
CNS	4,345	71.9	2,552	47.5	53.1	2,558	49.3	13.6
Autoimmune/ Inflammation	4,381	69.0	2,378	42.3	47.8	1,918	61.3	13.9
Genitourinary	686	67.7	421	55.2	59.1	417	64.6	21.0
Infectious Disease	3,553	69.4	1,996	46.0	60.4	2,251	77.3	25.6
Ophthalmology	630	86.0	416	61.5	61.5	727	73.8	31.3
Vaccines (Infectious Disease)	1,700	76.6	1,103	42.4	60.0	1,069	87.5	34.8
Overall	36,060	66.9	18,014	38.0	49.6	21,394	58.8	13.2

Trials originating from clinicaltrials.gov only								
	Phase 1 to Phase 2		Phase 2 to Phase 3			Phase 3 to Approval		Overall
Therapeutic Group	Total Paths	$POS_{1,2}$, %	Total Paths	$POS_{2,3}$, %	$POS_{2,APP}$, %	Total Paths	$POS_{3,APP}$, %	$POS_{1,APP}$, %
Oncology	13,437	61.2	5,128	32.3	4.9	888	28.3	2.6
Metabolic/ Endocrinology	2,417	81.3	1,651	61.1	21.4	745	47.4	19.2
Cardiovascular	1,831	81.0	1,310	69.0	29.1	679	56.1	26.6
CNS	3,076	79.5	2,012	54.0	17.4	763	45.9	15.1
Autoimmune/ Inflammation	3,114	74.5	1,781	50.2	18.6	597	55.6	14.6
Genitourinary	477	74.4	320	60.0	30.0	144	66.7	24.4
Infectious Disease	2,805	72.8	1,651	61.8	36.0	790	75.2	27.2
Ophthalmology	514	90.1	358	62.0	34.1	164	74.4	34.8
Vaccines (Infectious Disease)	1,371	77.8	887	60.2	44.4	453	87.0	35.5
Overall	29,042	70.1	15,098	49.8	19.0	5,225	55.0	13.4

Table S26. The probability of success by therapeutic group using truncated datasets. The top half shows the results using only trials between January 1, 2006, and October 31, 2015. The bottom half shows the results using only trials tagged as originating from *clinicaltrials.gov*.
